# Site-specific randomization of the endogenous genome by a regulatable CRISPR-Cas9 *piggyBac* system in human cells

**DOI:** 10.1038/s41598-017-18568-4

**Published:** 2018-01-10

**Authors:** Kentaro Ishida, Huaigeng Xu, Noriko Sasakawa, Mandy Siu Yu Lung, Julia Alexandra Kudryashev, Peter Gee, Akitsu Hotta

**Affiliations:** 1 Department of Life Science Frontiers, Center for iPS cell Research and Application (CiRA), Kyoto University, Kyoto, Japan; 2 Core Center for iPS Cell Research, Research Center Network for Realization of Regenerative Medicine, Japan Agency for Medical Research and Development (AMED), Tokyo, Japan; 3 Massachusetts Institute of Technology, Cambridge, Massachusetts USA; 4 Institute for integrated Cell-Material Sciences (iCeMS), Kyoto University, Kyoto, Japan

## Abstract

Randomized mutagenesis at an endogenous chromosomal locus is a promising approach for protein engineering, functional assessment of regulatory elements, and modeling genetic variations. In mammalian cells, however, it is challenging to perform site-specific single-nucleotide substitution with single-stranded oligodeoxynucleotide (ssODN) donor templates due to insufficient homologous recombination and the infeasibility of positive selection. Here, we developed a DNA transposon based CRISPR-Cas9 regulated transcription and nuclear shuttling (CRONUS) system that enables the stable transduction of CRISPR-Cas9/sgRNA in broad cell types, but avoids undesired genome cleavage in the absence two chemical inducing molecules. Highly efficient single nucleotide alterations induced randomization of desired codons (up to 4 codons) at a defined genomic locus in various human cell lines, including human iPS cells. Thus, CRONUS provides a novel platform for modeling diseases and genetic variations.

## Introduction

Among the millions of known genetic variations, single nucleotide variations (SNVs) are important because they account for more than half of all disease-causing mutations^1^. Likewise, to model diseases and investigate the consequences of genetic variations, cultured human cells are valuable research tools to mimic *in vivo* cell types. In particular, human embryonic stem (ES) cells and induced pluripotent stem (iPS) cells have been used broadly to model genetic diseases, owing to their capacity for unlimited self-renewal and ability to differentiate into a wide variety of cultured cell types^2,3^.

Recent advances utilizing the bacteria derived adaptive immune system, CRISPR (clustered regularly interspaced short palindromic repeats)-Cas9 (CRISPR associated protein 9), has enabled site-specific DNA cleavage to induce double strand breaks (DSBs)^4^. The DNA damage caused by DSBs immediately triggers one of two major DNA repair pathways: non-homologous end joining (NHEJ) to induce deletions or insertions, and homologous recombination (HR) to induce targeted insertion or base substitution by supplying an appropriate donor template. However, the transduction efficiency of human cells is low in general, and only a subset of cells can be transfected with Cas9, sgRNA and donor DNA templates. In addition, because HR occurs less frequently than NHEJ in mammalian cells, enhancing HR events has been a major challenge in the genome editing field^5^.

Accordingly, numerous groups have developed various techniques to improve HR frequency and to isolate genome-edited clones. Traditionally, the knock-in of a selection cassette (i.e. drug resistance gene, fluorescent gene, or enzyme) has been utilized to identify and enrich a rare cell population. The selection cassette is subsequently removed by Cre-loxP mediated recombination, *piggyBac* transposon based foot-print-free excision^6^, or site-specific nuclease mediated excision^7^. However, targeting and removal processes require two rounds of subcloning, which is labor intensive for establishing genome-edited cells. Instead of a double-stranded DNA template^8^, single-stranded DNA or single-stranded oligodeoxynucleotides (ssODNs) can serve as a donor to introduce a single nucleotide substitution^9^. Owing to easier construction and simpler use, ssODN mediated nucleotide substitution is a preferred technique for single nucleotide substitutions, but drug-selection cannot be utilized due to the synthesis limit of the donor template (typically a few hundred bases). Hence, it is necessary to perform extensive screening of subclones^10^, or sib-selection methods using droplet digital PCR^11^ to enrich rare populations. To enhance HR frequency, the optimization of ssODN donor design^12,13^, chemical modification of ssODN^14^, or chemical inhibitors^15–17^ (see also Supplementary Table 1) have also been reported.

Improved HR efficiency by ssODN donor templates has also been demonstrated in ES/iPS cells using efficient and conditional genome editing systems based on the inducible expression of Cas9 (iCRISPR)^18–20^. However, establishing the iCRISPR system initially requires a full round of genome editing to introduce the Dox-inducible Cas9 cassette into a safe harbor (i.e. AAVS1) locus. This step is time-consuming and laborious, making it difficult for novices to apply this strategy to a variety of cultured cell lines.

Here, we report an improved *piggyBac* DNA transposon vector to simplify the establishment of cells which stably express regulatable Cas9 for highly efficient and conditional genome editing. To avoid undesired background cleavage, methods to control Cas9 activity using 4-HT inducible inteins^21^, rapamycin inducible dimerization^22^, or blue-light inducible photoactivation^23^ have been employed. In our system, Cas9 is temporally regulated by a doxycycline-inducible TetO promoter^18–20,24^ in combination with spatial regulation by a steroid hormone receptor for nuclear shuttling^25^ to minimize background cleavage. By utilizing our CRONUS (CRISPR-Cas9 regulated by transcription and nuclear-shuttling) system and an appropriate ssODN template, we show highly efficient single nucleotide editing in human cells, including iPS cells. Owing to a very high nucleotide substitution rate via HR, we further demonstrate codon shuffling at the *DMD* gene locus, which is associated with Duchenne muscular dystrophy^26^, and the *HLA-A* gene locus, which is a well-known gene that shows large genetic variations between individuals.

## Results

### *piggyBac* delivery of dual regulated CRISPR-Cas9 system

**Figure 1:**
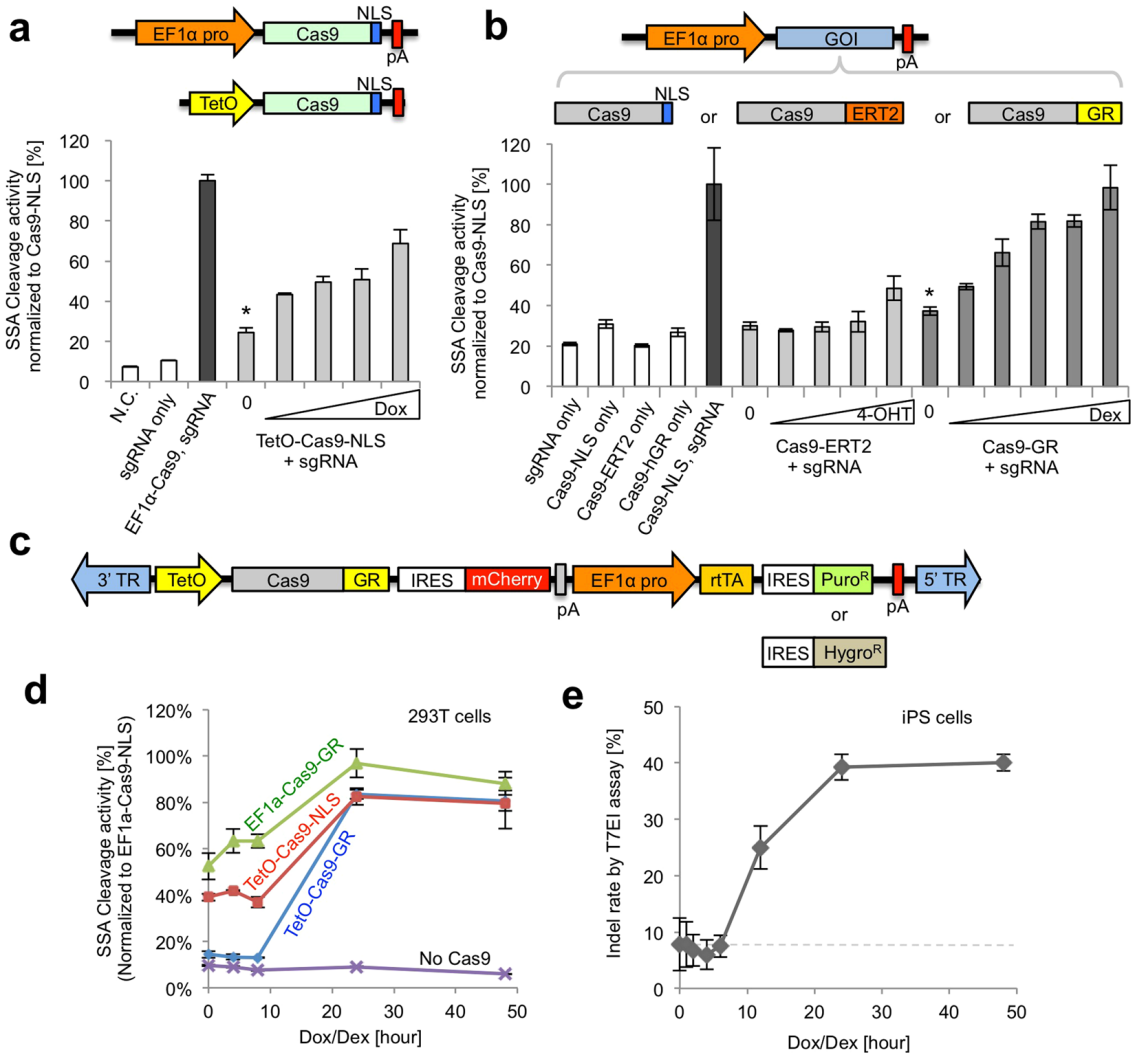
Development of CRISPR-Cas9 regulated by transcription and nuclear shuttling (CRONUS) system in a *piggyBac* vector. (**a**) Cas9 activity is regulated by Dox in a dose dependent manner. A *piggyBac* vector expressing SpCas9 with SV40 NLS (nuclear localization signal) and rtTA (reverse tetracycline trans-activator) was transfected into 293 T cells and treated with various concentrations of Dox (0.0065, 0.065, 0.65, 6.5 μM). DNA cleavage activity was measured by SSA-Luc reporter activity. The asterisk indicates significant background cleavage activity. (**b**) SpCas9 fused with NLS, ERT2 (estrogen receptor variant), or GR (glucocorticoid receptor) was inserted into the EF1α-promoter derived *piggyBac* vector. Posttranslationally controlled SpCas9 was transfected into 293 T cells and treated with various concentration of 4-OHT (0.025, 0.25, and 2.58 μM) or Dex (0.00375, 0.015, 0.06, 0.25, and 1 μM). The GR/Dex system showed better regulation of SpCas9 activity compared with the ERT2/4-OHT system. Data are presented as the mean ± standard deviation from biologically independent samples (n = 3). The asterisk indicates significant background cleavage activity. (**c**) Vector design of the CRONUS *piggyBac* vector with the puromycin selection cassette or hygromycin selection cassette. 5′ and 3′ TRs (terminal repeats) of *piggyBac* transposon were derived from *Trichoplusia ni*. TetO promoter has multiple (×8) rtTA binding sites. IRES: internal ribosome entry site from encephalomyocarditis virus. (**d**) Time course experiment shows that CRONUS system (TetO-Cas9-GR) has a bigger induction rate of Cas9 activity compared with TetO-Cas9 or Cas9-GR single regulation systems when treated with 2 μM Dox and 1 μM Dex. Cas9 activity reached a plateau by 24 hours post-treatment. (**e**) Time-course assay of the genome cleavage activity of the CRONUS iPS cells upon the addition of Dox and Dex. The background signal of the T7EI assay (an average from 3 independent experiments) is indicated by a gray dotted line. Data are presented as the mean ± standard deviation from technically independent samples (n = 3).

We first sought to enhance the genome editing efficiency in human cells by stably expressing the CRISPR-Cas9 transgene into target cells, which avoids suboptimal transduction. At the same time, Cas9 DNA cleavage activity should be regulated to avoid undesired mutagenesis. To develop a regulatable Cas9 expressing vector, we first tested several *piggyBac* vector constructs in 293T cells by transient transfection. We chose the *piggyBac* transposon vector because it can efficiently integrate into the genome in various cell types, with a much larger packaging capacity compared with other integrating vectors, such as retroviral/lentiviral vectors. We initially constructed a doxycycline (Dox)-inducible Cas9-expressing *piggyBac* vector similar what has been recently reported^24^ and investigated the genome editing efficiency at exon 45 of the human *DMD* gene by a luciferase-based single strand annealing (SSA) assay in 293T cells (Fig. 1a). We observed dose dependent Dox-inducible activity of the Cas9-mediated target cleavage, however, small but considerable background cleavage activity was observed even without Dox. We suspect this is due to leakiness of the TetO promoter, as we utilized regular fetal bovine serum which normally contains a low level of tetracycline. Next, we fused a variant of estrogen receptor (ERT2)^27^ or glucocorticoid receptor (GR) with Cas9 to investigate ligand dependent nuclear shuttling of Cas9 upon binding with Z-4-hydroxytamoxifen (4-OHT) or dexamethasone (Dex), respectively. We observed substrate-dependent activation of the Cas9 cleavage activity with GR-Dex, however, the background cleavage activity was significantly higher than those of negative controls (Fig. 1b **)**.

Because neither the Dox- nor Dex-inducible vector alone showed high background cleavage even in the absence of chemical ligands, we combined both elements into one vector to minimize this background activity (Fig. 1c). As a result, the background level using the new combined vector was dramatically reduced to a level similar to those of the negative controls, but the cleavage activity could be rapidly induced upon the addition of both Dox and Dex (Fig. 1d). Although high background cleavage was observed even in the absence of Dox/Dex (0 hour) in vectors regulated by GR-Dex (EF1α-Cas9-GR) or TetO-Dox (TetO-Cas9-NLS), our dual regulated system (TetO-Cas9-GR) showed a reduced background level similar to that of the control without Cas9 (Fig. 1d). Importantly, from 8 to 24 hours, a sharp induction of Cas9 cleavage activity was observed with the TetO-Cas9-GR dual regulated *piggyBac* vector, demonstrating efficient induction by combining the two regulatory components. We named our *piggyBac* vector CRONUS (CRISPR-Cas9 regulated by transcription and nuclear-shuttling).

**Figure 2:**
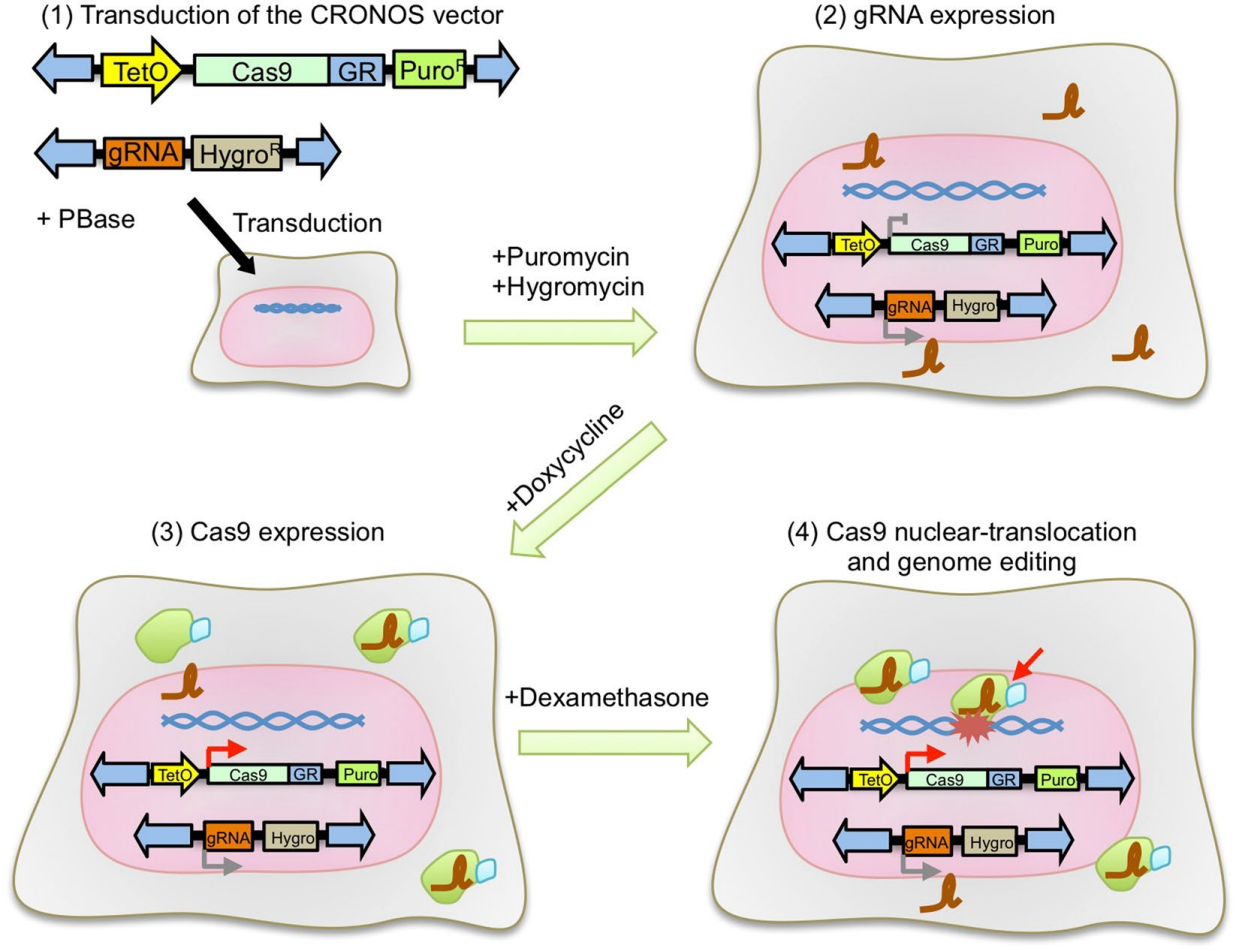
Establishment of CRONUS iPS cells and functional assessments. Schematic drawing of the CRONUS system. (1) First, the CRONUS *piggyBac* vector (PuroR) was transfected into target cells along with the gRNA expressing *piggyBac* vector (HygroR) and *piggyBac* transposase (PBase). (2) Upon puromycin and hygromycin selection of the transfected cells, the CRONUS and sgRNA expression cassettes are stably integrated into the chromosome of the target cells. (3) The addition of Dox activates Cas9-GR expression. (4) The addition of Dex induces the nuclear -translocation of Cas9-GR, where it binds and cleaves on target genomic DNA.

To test the utility of the CRONUS system for non-homologous end joining (NHEJ) mediated insertion or deletion (indel) induction in human iPS cells, we introduced CRONUS together with a sgRNA-expressing *piggyBac* vector. Stable integration of the Cas9 and sgRNA vectors was carried out with puromycin and hygromycin, respectively (Fig. 2). Then, we monitored genome editing efficiency after Dox/Dex treatment by T7EI assay (Fig. 1e). Genome cleavage was detectable 6 hours after Dox/Dex treatment and reached a plateau by 24 hours. NHEJ mediated genome cleavage activity was over 40% at the *DMD* gene locus, which is similar to the previously reported iCRISPR system (24–57% for single sgRNA at various gene loci) that expresses Cas9 from the AAVS1 locus^18^.

### Enhancement of ssODN-mediated single nucleotide alteration

To functionally assess the impact of single nucleotide variations (SNVs), ssODN-mediated knock-in is a powerful tool, however, the frequency of HR events in human cells is low and screening a large number of subclones is a major bottleneck. To optimize ssODN-mediated targeting, we first developed a sensitive screening method by using two sets of primers specific for detecting modified and non-modified alleles, respectively, to quantify the allele frequency by qPCR (Fig. S1a) Although the allele-specific PCR method can not distinguish whether the allele is perfectly targeted or includes additional indels, it is much quicker than conventional subcloning and sequencing assays. Also, we confirmed that, with our primer design, we could sensitively and quantitatively detect the copy number of the targeted allele as low as 0.1% compared with the non-edited allele (Fig. S1b).

By using this assay system, we first tested transient transfection of Cas9 and sgRNA (targeting the *DMD* gene) expressing plasmid DNAs in combination with suppression of the NHEJ pathway by siRNAs (targeting KU70, KU80, or LIG4)^16^ (Fig. S1c,d), but in our hands, no obvious enhancement was detected, partially due to cellular damage of repeated transfections to first deliver siRNA and, later, the plasmid DNA. Next, we tried chemical compounds such as L755507 and Brefeldin A, since they have been reported to enhance the rate of HDR^15^. We also tested YM155, which is a survivin suppressor and known to induce the DNA damage response pathway. However, no obvious enhancement was detected (Fig. S1e).

**Figure 3:**
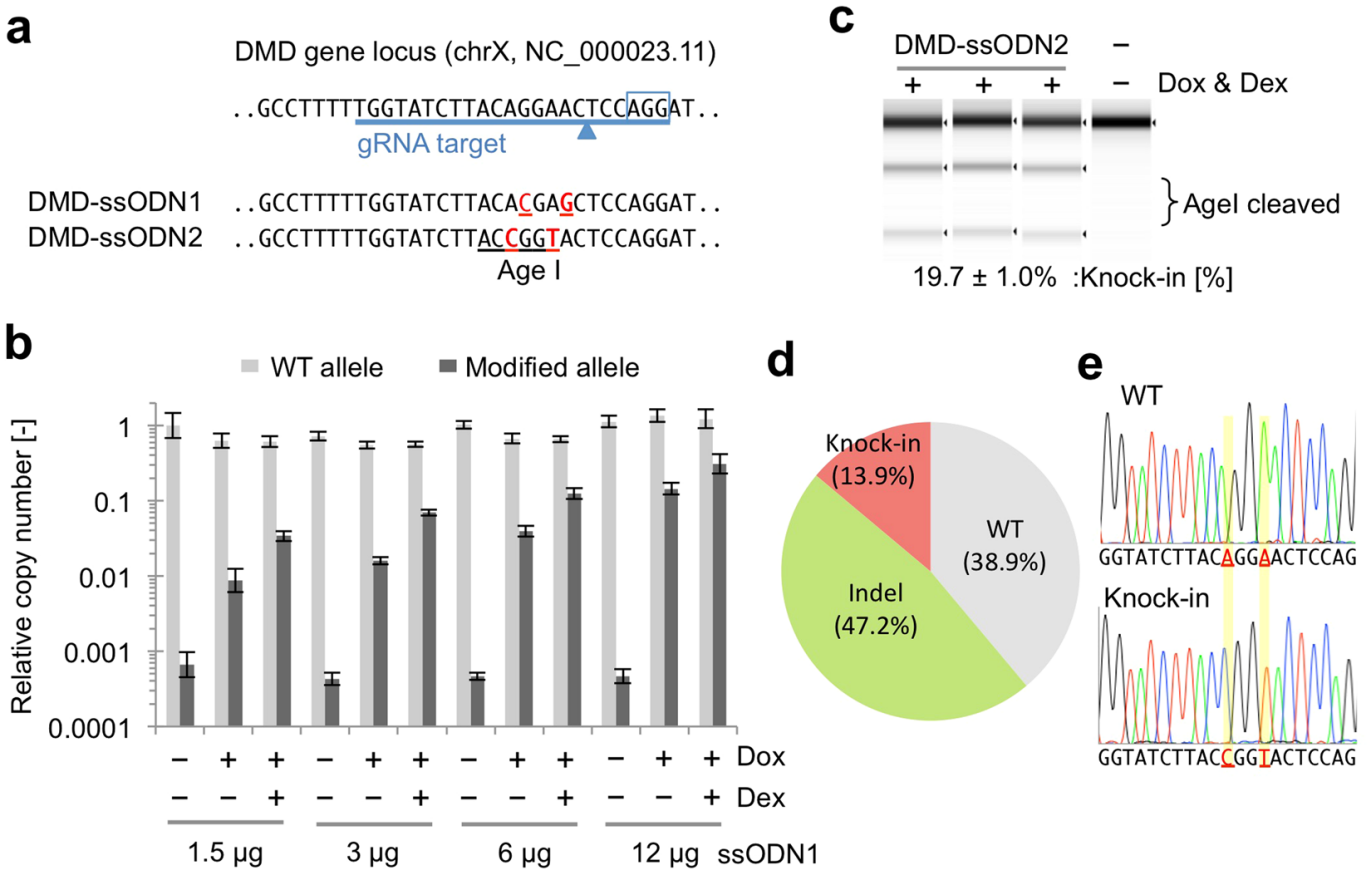
Optimization of ssODN-mediated targeting of the *DMD* gene locus by the CRONUS system. (**a**) Experimental design on the *DMD* gene target. The gRNA target sequence with PAM (boxed) and Cas9 cleavage site (arrowhead) is indicated alongside two ssODN donor templates (100-mer each). Intended substitution sites are marked in red, and a *de novo* AgeI restriction enzyme site is underlined. (**b**) Optimization of the amount of ssODN for knock-in experiments. CRONUS iPS cells were transfected with DMD-ssODN1 and treated with Dox and Dex for 48 hours. Knock-in efficiency was measured by allele-specific qPCR. Relative copy number of the non-altered WT allele is indicated by gray bars, and modified allele specific copy number is indicated by black bars. Mean ± standard deviation. (**c**) CRONUS iPS cells were transfected with DMD-ssODN2 and treated with Dox and Dex for 48 hours. Knock-in efficiency was measured by the band intensities of AgeI cleaved bands by the RFLP assay. (**d**) CRONUS iPS cells were transfected with DMD-ssODN2 and treated with Dox and Dex for 48 hours. Subclones were isolated, and each clone was genotyped by TA-cloning and Sanger sequencing. Only intact sequences with expected knock-in clones are classified and counted as “knock-in” clones. (**e**) Representative sequence electrograms of WT and knock-in clone at the target site.

Since we could induce an extremely high level of NHEJ by using CRONUS in iPS cells, we reasoned that CRONUS would also enhance the efficiency of ssODN-mediated HRs. To this end, we transfected an ssODN template into a male iPS cell line (containing a single copy of the *DMD* gene) using 4D-Nucleofector (Fig. 3a) in the presence or absence of Dox and Dex and then quantified the genomic copy number of modified *DMD* gene alleles by qPCR. To our surprise, a very high frequency of knock-in events was observed upon Dox/Dex treatment, which was clearly dependent on the amount of ssODN transfected (Fig. 3b). We further validated the knock-in by an RFLP assay using the restriction enzyme AgeI, which only cleaves when ssODN-mediated targeting is successful and retains the 6-bp target sequence intact. Approximately 19.7% of the PCR product was successfully cleaved by AgeI, clearly demonstrating the successful knock-in of the ssODN donor (Fig. 3c). Finally, we assessed the frequency of knock-in by Sanger sequencing of the subclones and found that 47.2% (34/72) of the sequence reads showed random indels, which were presumably generated by NHEJ, whereas 13.9% (10/72) of the clones showed successful knock-in without any indels (Fig. 3d,e and Fig. S2a).

**Figure 4:**
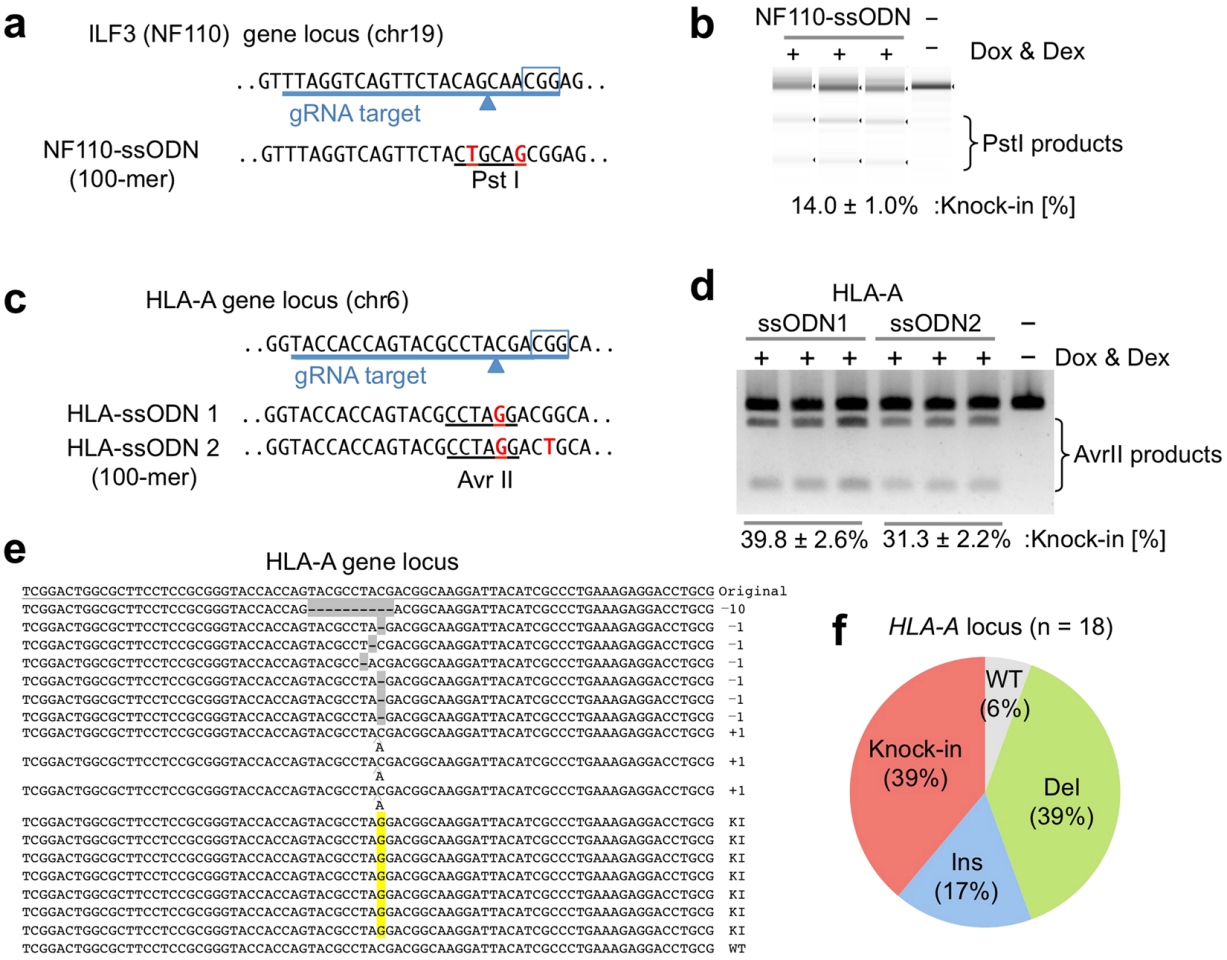
Optimization of ssODN-mediated targeting of *ILF3* and *HLA-A* gene loci by the CRONUS system. (**a**) Design of sgRNA that targets the *ILF3* gene on chromosome 19 and ssODN with two nucleic acid alterations (red) overlapping the PstI restriction enzyme site (underlined). The PAM sequence is boxed, and the Cas9 cleavage site is indicated by the arrowhead. (**b**) Knock-in efficiency was measured by the RFLP assay using PstI restriction enzyme. (**c**) Design of sgRNA targeting the *HLA-A* gene on chromosome 6 and two ssODN templates. Intended substitution sites are marked in red, and *de novo* AvrII restriction enzyme site is indicated by underline. HLA-ssODN2 has a blocking mutation in the PAM sequence, whereas HLA-ssODN1 does not. (**d**) Knock-in efficiency was measured by the RFLP assay using AvrII restriction enzyme. (**e**) The Sanger sequence of each subclone confirmed successful knock-in in 7 clones out of 18 clones analyzed. (**f**) The breakdown of each sequence in (**e**) is indicated by the pie chart.

We further targeted additional gene loci, *ILF3* on chromosome 19, which is a double-stranded RNA binding protein associated with ovarian cancer, and we observed about 14% of knock-in events in the *ILF3* gene (Fig. 4a,b). In addition, we tested whether the CRONUS system can be used to target an allele-specific sequence by targeting one allele of the *HLA-A* gene on chromosome 6, which is one of the best examples of genetic variation among individuals. We designed a sgRNA which targets the HLA-A*02:07 sequence, but differs by two nucleotides from the other allele of the HLA-A*32:01 sequence (Fig. S5c). With this locus, we observed even higher knock-in events (over 30%) when analyzed by the RFLP assay using AvrII restriction enzyme (Fig. 4c), and we validated the allele specific targeting by utilizing allele-specific PCR amplification and sequencing of the subclones (Fig. 4e,f).

To test the functional pluripotency of the iPS cells harboring a CRONUS *piggyBac* vector, we performed embryoid body (EB) differentiation of the original 1383D2 iPS cells and CRONUS-1383D2 iPS cells and monitored the expression levels of the pluripotent marker gene NANOG. As expected, NANOG expression was rapidly down-regulated after 10 days of EB differentiation (Fig. S2b).

By taking advantage of our inducible Cas9 system, we assessed the time window of Cas9 induction relative to the electroporation timing of ssODN (Fig. S2c). As shown in Fig. S2d, Cas9 expression needed to be induced 12 hours prior or after the ssODN electroporation to achieve successful targeting via HDR.

A high level of Cas9/sgRNA expression and indel events prompted us to investigate the risk of off-target mutagenesis within the human genome for DMD sgRNA (Fig. S3a), ILF3 sgRNA (Fig. S4a), and HLA-A sgRNA (Fig. S5a). Among potential off-target sites predicted by the CRISPOR web tool using the CFD off-target score^28^, mutagenesis was undetectable by the T7EI assay under Dox and Dex treatment (Figs S3b, S4b and S5b,c). Additionally, no background genome cleavage was observed at the on-target site even after several passages in the absence of Dox and Dex treatments (Fig. S3c), suggesting that high specificity and tight regulation of the CRISPR-Cas9 system is maintained in our CRONUS system.

### Shuffling of codon sequences at a defined genomic locus

**Figure 5:**
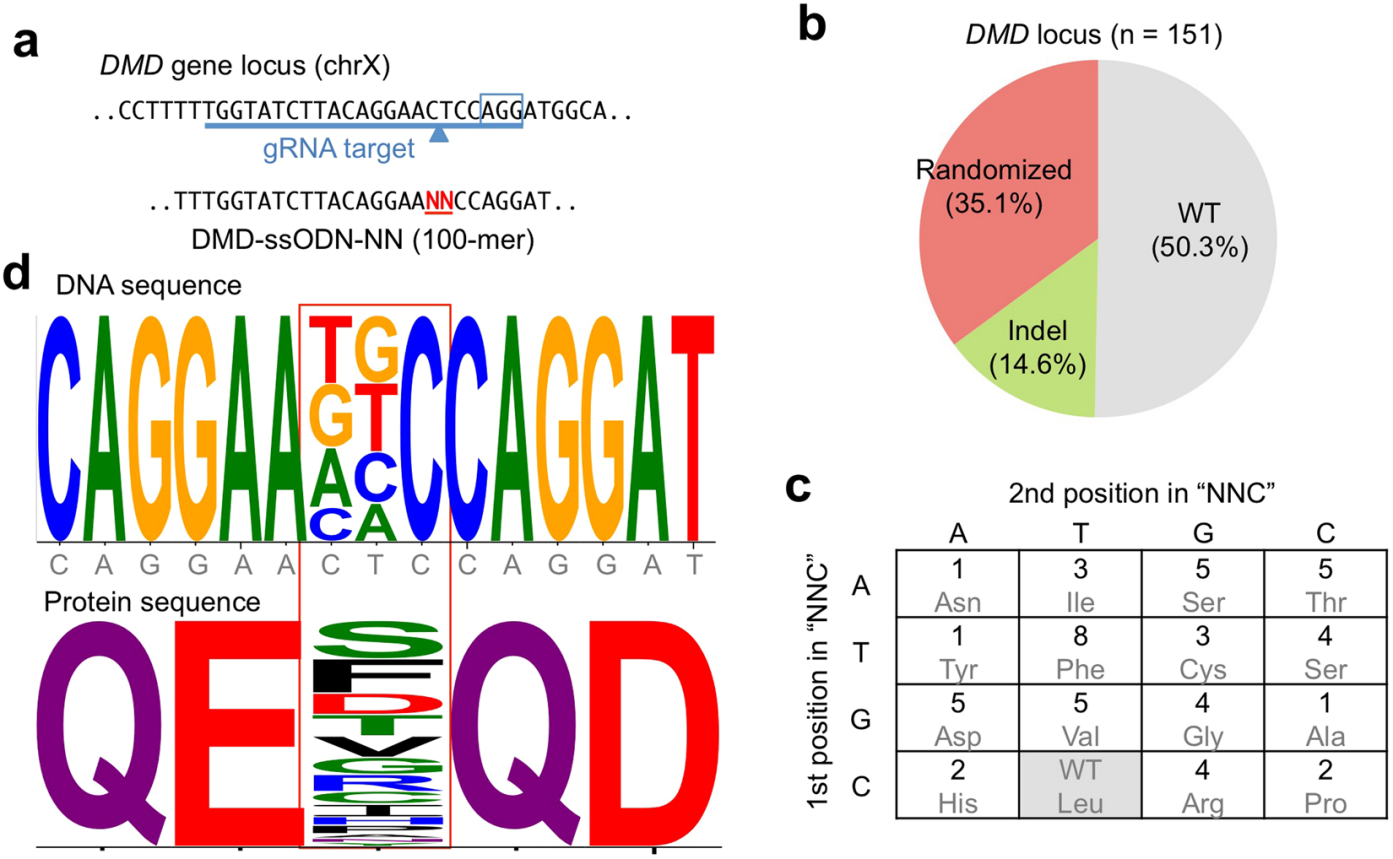
Site-specific shuffling of a single codon sequence at *DMD* gene locus in iPS cells. (**a**) Design of ssODN (100-mer) to induce randomization of a single amino acid at exon 45 of the *DMD* gene as “NNC”, where “N” is any of “A”, “T”, “C”, and “G” bases. The 3rd “C” was fixed to avoid the stop codon. The gRNA-targeting sequence is indicated by the blue underline. The PAM sequence is boxed, and the Cac9 cleavage site is indicated by the arrowhead. (**b**) Site-directed random mutagenesis at the *DMD* gene locus in human iPS cells 1383D2 line. Randomized ssODN was electroporated into CRONUS iPS cells and treated with Dox and Dex for 1 day. The genomic sequence of each subclone was analyzed by Sanger sequencing, and the breakdown of each sequence is indicated by the pie chart. Only clones without additional indel or other mutations are classified as “randomized”. See also Fig. S5. (**c**) Breakdown of each randomized clone with the corresponding amino acid codon. (**d**) Consensus sequence of the DNA and amino acid sequences of the randomized clones.

**Figure 6:**
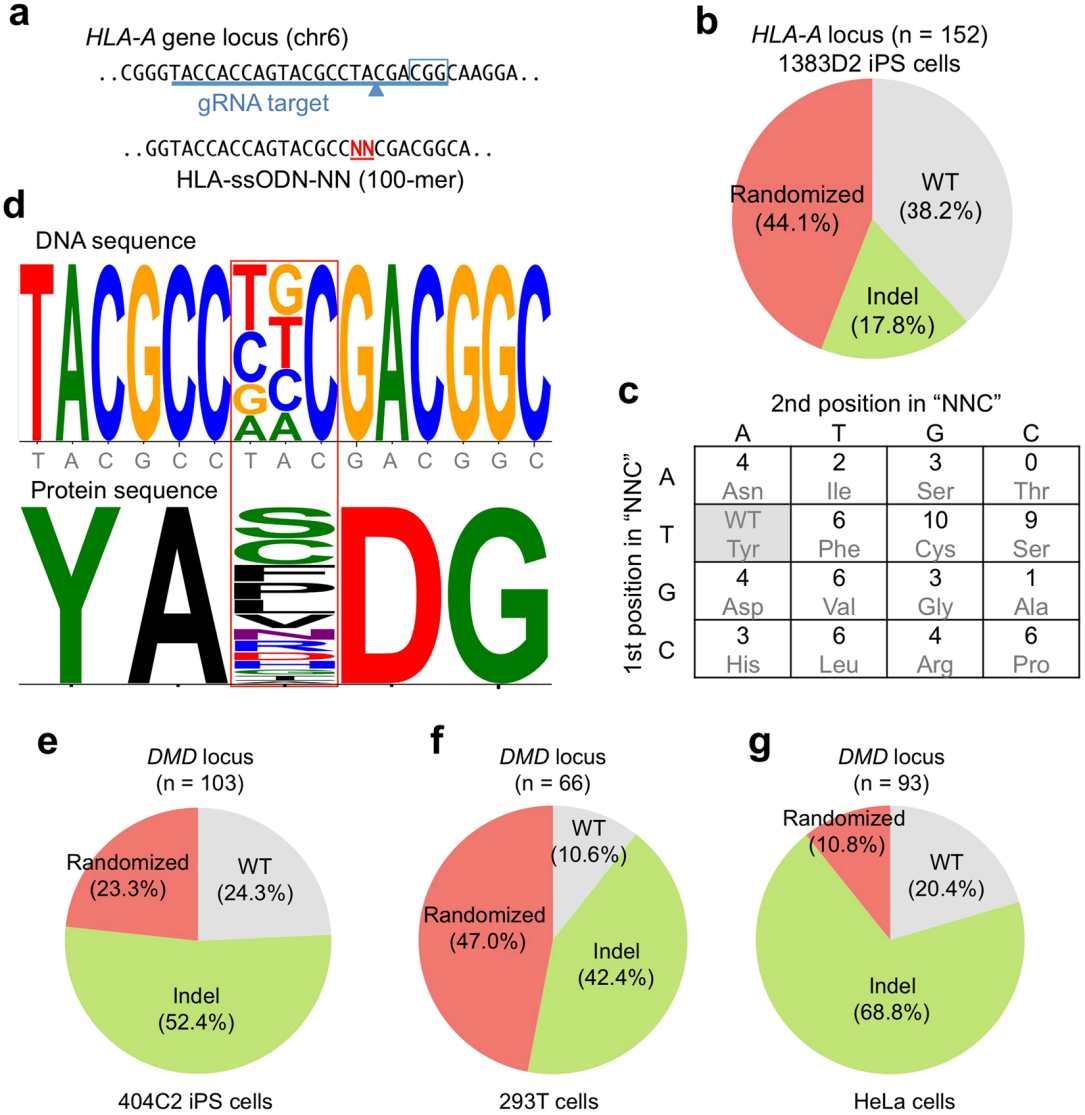
Site-specific shuffling of a single codon sequence at *HLA-A* gene locus in iPS cells. (**a**) Design of sgRNA that targets exon 3 of the *HLA-A* gene on chromosome 6 and randomized ssODN. The PAM sequence is boxed, and the Cas9 cleavage site is indicated by an arrowhead. (**b**) Site-directed random mutagenesis at the *HLA-A* gene locus in human iPS cells 1383D2 clone. Randomized ssODN was electroporated into iPS cells that were subsequently treated with Dox and Dex for 1 day. The genomic sequence of each subclone was analyzed by Sanger sequencing, and the breakdown of each sequence is indicated in the pie chart. See also Fig. S6. (**c**) Breakdown of each randomized clone with the corresponding amino acid codon. (**d**) Consensus sequence of the DNA and amino acid sequences of the randomized clones. (**e**–**g**) Site-directed random mutagenesis at the *DMD* gene locus in human 404C2 iPS cells, human embryonic kidney 293 T cells, or HeLa cells.

Taking advantage of CRONUS to induce a high frequency of ssODN-mediated HR, we sought to induce site-directed codon shuffling in human iPS cells. We electroporated CRONUS iPS cells with randomized ssODNs at two nucleotide positions on the *DMD* gene locus to shuffle a codon sequence (from “CTC” to “NNC”) without generating a stop codon (Fig. 5a). Among the 151 subclones analyzed, 53 clones (53/151 = 35.1%) showed expected randomization at the defined two nucleotides without additional indels (Fig. 5b and Fig. S6). This resulted in the generation of all the possible 16 codon sequences (Fig. 5c). Interestingly, no bias was observed between single nucleotide alterations (“CTC” to “CNC” or “NTC”, 24 clones) vs. two nucleotide alterations (“NNC”, 29 clones) (Fig. 5d). Moreover, we also introduced the same approach on the *HLA-A* gene locus and again found a similar or even greater level of knock-in events, in which 67 clones had been successfully randomized out of 152 clones analyzed (67/152 = 44.1% success rate) (Fig. 6a–d and Fig. S7).

To test the applicability of the CRONUS system in other human cell lines, we introduced the CRONUS *piggyBac* vectors into 404C2 iPS cells, HEK293T cells and HeLa cells (Fig. 6e–g **)**. Even though the same sgRNA targeting the *DMD* locus was used, we found that the targeting efficiency varied between different cell lines, suggesting different chromatin status and/or DNA repair activity might play a role in modulating the targeting events. Nonetheless, our data demonstrate that the CRONUS *piggyBac* vector system can be applicable for various cell types to induce a high degree of nucleic acid shuffling at a desired locus.

**Figure 7:**
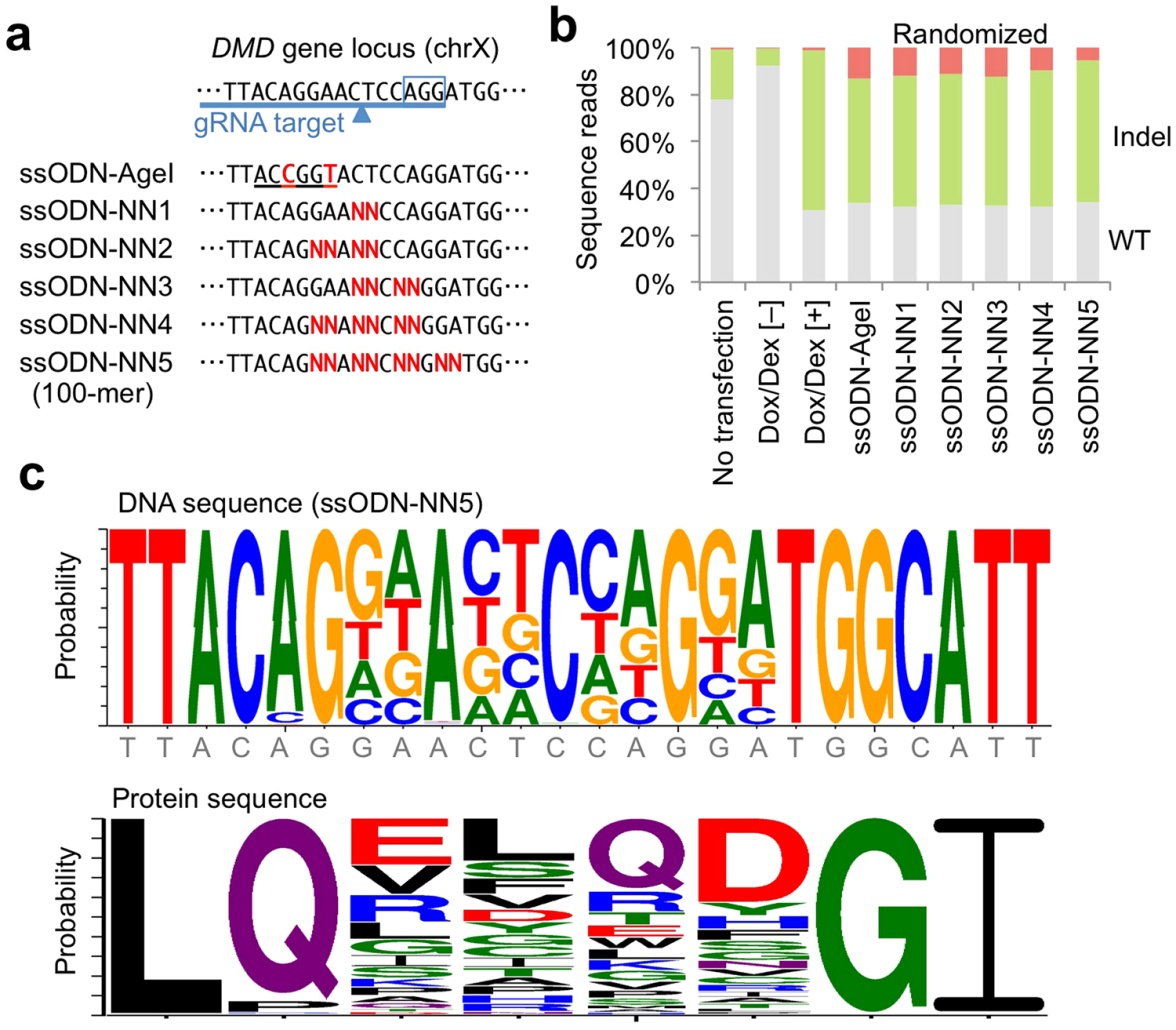
Site-specific shuffling of multiple codon sequences at *DMD* gene locus in iPS cells. (**a**) Design of ssODN (100-mer) to induce randomization of up to four amino acids at the *DMD* gene locus. ssODN-AgeI (same as ssODN-DMD2 in Fig. 3a) was used as a control to monitor the knock-in efficiency. (**b**) ssODN donor template with multiple randomization sites was electroporated into CRONUS iPS cells and treated with Dox and Dex for 1 day. The genomic sequence from the bulk cell population was analyzed by deep sequencing, and the breakdown of each sequence read is indicated by the bar graph. Sequence reads perfect matching the original sequence were classified as “WT (wild-type)”, and sequence reads with any indel or mutation were labeled as “indel”. Only sequence reads without indel or mutations other than the expected randomized site(s) are classified as “randomized”. (**c**) Consensus sequence of the DNA and amino acid sequences of the randomized sequence reads that were treated with ssODN-NN5.

Lastly, we investigated the number of bases that can be shuffled simultaneously with our CRONUS system. For this experiment, we designed five ssODNs with multiple shuffling sites, up to 8 bases (4 codons) around the Cas9 cleavage site (Fig. 7a). After electroporation of each ssODN, bulk collected cells were analyzed by amplicon deep sequencing. Interestingly, administration of Dox and Dex induced greater than 60% indels in the absence of ssODN template, whereas some indel events were substituted by base replacement when a ssODN-AgeI or ssODN-NN donor was supplied. The base substitution (randomized) rate was highest with ssODN-AgeI and ssODN-NN1, and was gradually decreased inversely with the number of the randomized bases in the ssODN donor (Fig. 7b). Although the randomized rate was lowest with the ssODN-NN5 donor template, we confirmed that all 8 bases designed as “N” were successfully incorporated into the genomic sequence we analyzed (Fig. 7c). Therefore, up to 4 codon sites can be simultaneously randomized by the CRONUS system.

## Discussion

In summary, we developed a novel genome editing platform that we termed CRONUS, which utilizes a *piggyBac* transposon vector for the stable integration of a dual regulated CRISPR-Cas9 system into genomes. CRONUS enables the simple, cost-effective and rapid establishment of cell lines in which highly efficient genome editing can be performed upon the simple addition of two chemical compounds into culture media. By eliminating the need to supply Cas9 and sgRNA exogenously, an extremely high degree of single nucleotide alterations (>10% knock-in events) can be achieved using ssODN donor templates without the need for drug selection. This feature is important in a practical sense because a knock-in frequency greater than 10% indicates that only 10 cell clones would need to be screened to isolate one positive clone versus screening several hundred clones if the knock-in frequency were less than 1%. We anticipate that highly efficient nucleotide alteration might be possible with other inducible Cas9 systems integrated into a safe harbor, such as the iCRISPR system, but our CRONUS *piggyBac* system does not require the initial targeting and subcloning process to establish the stable cell line, hence it would facilitate the use of genome editing for researchers who are novices in the field.

Interestingly, despite nearly 100% of the cells expressing both Cas9 and sgRNA at a high-level, genome editing efficiency did not reach greater than 50% when measured by the T7EI assay. Of note, prolonged induction of Cas9 for up to 48 hours did not increase the genome editing rate (Fig. 1e), suggesting the system already reached a saturation point, which is similar to the previously reported Dox-inducible Cas9 system (i.e. iCRISPR). One possible explanation might be that the host DNA repair pathway is actively correcting the sequence precisely after DNA cleavage. However, the reason for the incomplete genome editing is yet to be explored.

We further demonstrated that our CRONUS system enables site-specific random mutagenesis in endogenous human genomes. This feature too has several advantages over traditional *in vitro* enzymatic or chemical approaches^29^, because the influence of the mutagenesis can be readily detected in the native genomic environment. The current standard in genome editing typically involves the introduction of a single nucleotide substitution one at a time after extensive screening, whereas our method can generate multiple lines with different sequences simultaneously, including the original cell line as an isogenic control. Hence, our approach would facilitate the interrogation of the functional significance of SNVs identified by genome-wide association studies, including tri-allelic SNPs^30^. Furthermore, if the same approach is applied to a protein coding sequence, one could shuffle amino acid sequences at a defined locus for saturation mutagenesis. Thus, our approach would offer an unprecedented methodology for engineering endogenously expressed human proteins to alter functionality, stability and to understand molecular mechanisms in various cell types. Alternatively, nucleic acid sequence could be shuffled without altering the amino acid sequence (i.e. silent mutation) to assess the impact of nonsense mutations^31,32^. Due to the complexity of bi-allelic targeting, we propose to target a haploid gene (such as the *DMD* gene on the X chromosome in male cells or HLA-A gene utilizing allele specific SNVs) or to employ “gain-of-function” screening where single allelic targeting would gain a cellular phenotype for subsequent selection and sequencing to identify the corresponding allelic sequences. Given the expanding demand for genome editing, we anticipate that our simple and cost-effective method will help to further model human genetic variations in culture by a broad range of scientists to understand human genetic traits and diseases.

## Methods

### Vector information

The details of the vector construction procedures are described in the Supplemental Methods section. The vectors and sequence information described in this study will be available on Addgene. Accession codes are: pPV-TetO-SphcCas9-GR-iC-A-EF1α-rtTA-iP (CRONUS- Puro, Addgene ID 100596), pPV-TetO-SphcCas9-GR-iC-A-EF1α-rtTA-iH (CRONUS-Hygro, Addgene ID 100597), pPV-H1-ccdB-mEF1α-RiH (*piggyBac* vector for sgRNA cloning, Addgene ID 100598), and pHL-EF1α-hcPBase-A (*piggyBac* transposase expression plasmid, Addgene ID 100599).

### Cell culture

Human dermal fibroblast (Cat. No. HDF1388, Female, Cell Applications, Inc.)-derived 404C2 iPS cells reprogrammed by episomal vectors^33^ or PBMC (Cat. No. CTL-CP1 LP_53, Donor #40, Male, Cellular Technology Ltd)-derived 1383D2 iPS cells reprogrammed by episomal vectors were cultured under feeder-free, xeno-free condition as previously described^34^. Briefly, human iPS cells were cultured on recombinant Laminin 511 E8 fragment (iMatrix-511, Cat. No. 892012, Nippi)-coated cell culture plates with StemFit AK03N medium (Ajinomoto). Before plating the cells, the plates were coated with iMatrix-511 (0.25–0.35 μg/cm^2^) and incubated overnight in a CO_2_ incubator (37 °C). Before cell passaging, iPS cells were dissociated into single cells by treatment with 0.5× TrypLE Select [1× TrypLE Select (Cat. No. 12563011, Thermo Fisher Scientific) diluted 1:1 with 0.5 mM EDTA/PBS] for 20 seconds at 37 °C. The splitting ratio of feeder-free iPSCs was between 1:3 and 1:20 every 2–5 days. ROCK inhibitor Y-27632 (Cat. No. 251-00514, Wako) was added to the culture medium at a final concentration of 10 μM on the day of and day after passaging. Cell cultures were routinely tested using MycoAlert Mycoplasma Detection Kit (Cat. No. LT07-218, Lonza) to ensure that cells were free of mycoplasma contamination.

HEK293T cells (Cat. No. CRL-3216, ATCC) were maintained in DMEM (Cat. No. 08459-64, Nacalai tesque) containing 10% fetal bovine serum (Cat. No. 10437-028, Gibco). For knock-in experiments, 1.5–1.8 × 10^6^ 293T cells in 6-well dishes were transfected using Lipofectamine 2000 transfection reagent (Cat. No. 11668019, Thermo Fisher Scientific) according to the manufacturer’s instructions. For each well, a total of 1 μg of expression plasmid containing Cas9 and sgRNA and 300 ng ssODN was used. For siRNA-mediated knockdowns in 293 T cells, 1.5–1.8 × 10^6^ cells were transfected with 5 pmol siRNA (Applied Biosystems) using Lipofectamine RNAiMAX (Cat. No. 13778030, Thermo Fisher Scientific) according to the manufacturer’s instructions. The siRNA sequences for transfection are shown in Supplementary Table 4. For knocking down the candidate factors in advance, we transfected siRNA two days prior to the transfection of the Cas9/sgRNA plasmid DNAs and the ssODN template. To inhibit the NHEJ pathway, L755507 (Cat. No. sc-204045, Santa Cruz Biotechnology), Brefeldin A (Cat. No. 022-15991, Wako), or YM155 (Cat. No. 781661-94-7, Calbiochem) was used. These drugs were added to the medium on the same day as Cas9/sgRNA plasmids and ssODN transfection as previously described^15^. We monitored the transfection efficiency by checking the mRFP expression from the pPV-H1-sgRNA-mEF1α-RiH vector and approximately 80% of the cells were RFP positive. Two days after plasmid and ssODN transfection, cells reached near confluency and were harvested for genotyping. It is worth noting that the cells treated with 10 μM of YM155 showed marked cellular toxicity by visual inspection using an inverted microscope.

To establish CRONUS iPS cell lines, 1 × 10^6^ cells were suspended in 100 μL of Opti-MEM I (Cat. No. 31985-070, Thermo Fisher Scientific) with *piggyBac* vectors (pPV-TetO-SphcCas9-iC-A-EF1α-rtTA-iP: 4 μg, pPV-H1-gRNA-mEF1α-RiH: 4 μg) and a *piggyBac* transpose vector (pHL-EF1α-hcPBase-A: 2 μg). Cells were then electroporated with a NEPA 21 electroporator (poring pulse: pulse voltage, 125 V; pulse width, 5 ms; pulse number, 2; Nepa Gene) in a 0.2 mm gap cuvette (Cat. No. EC-002, Nepa Gene). After electroporation, cells were plated onto one well of a 12-well iMatrix-511-coated plate in StemFit AK03N media with 10 μM Y-27632. Three days after electroporation, cells were allowed to grow to near confluency and split in a 6-well iMatrix-511-coated plate. The following day, cells were treated with 100–200 μg/mL of hygromycin B (Cat. No. 10687-010, Thermo Fisher) and 1–15 μg/mL of Puromycin (Cat. No. 29455-54, Nacalai tesque) for 5 days. To induce Cas9 activity, the following chemical compounds were used: 10 mM (3.88 mg/mL) of Z-4-Hydroxytamoxifen (Cat. No. H7904, Sigma-Aldrich) stock solution in ethanol, 10 mM (3.92 mg/mL) of Dexamethasone (Cat. No. 047-18863, Wako) stock solution in ethanol, and 10 mM (5.13 mg/mL) of Doxycycline (Cat. No. D5897, LKT Labs) stock solution in DMSO protected from light.

### Assessment of genome editing efficiency by SSA Luc assay

To transfect 293 T cells in a 96-well plate format, Firefly Luciferase reporter pGL4-SSA-DMD-all (100 ng), Renilla Luciferase transfection control phRL-TK (20 ng), Cas9 expressing vector (i.e. pPV-TetO-SphcCas9-iC-A-EF1α-rtTA-iP, 200 ng) and gRNA expressin vector (pPV-H1-gRNA-mEF1α-RiH, 200 ng) were diluted in 25 μL of Opti-MEM I Reduced Serum Medium (Thermo Fisher, Cat. No. 31985062). Next, 0.7 μL of Lipofectamine 2000 diluted in 25 μL of Opti-MEM I Reduced Serum Medium was mixed and incubated for 20–30 min at room temperature (20–25 °C). Then, tripsinized 293T cells were added to each well at 50,000 cells/100 μL/well. On the next day of transfection, we measured firefly and renilla luciferase activity by using Dual-Glo Luciferase Assay Kit (Cat. No. E2920, Promega) and LAS-3000 imaging system (Fujifilm). Firefly luciferase activity was normalized by renilla luciferase value and used as a readout of the target cleavage by Cas9 and corresponding gRNA.

### Assessment of genome editing efficiency by T7EI assay

The CRISPR-sgRNA target site was amplified by a high-fidelity PCR reaction from genomic DNA and purified by Wizard SV Gel and the PCR Clean up System (Promega). The primer sequences for PCR are shown in Supplementary Table 5. Purified PCR products (400 ng) were denatured (95 °C for 5 min) and re-annealed by gradually cooling from 95 °C to 85 °C at −2 °C/sec and 85 °C to 25 °C at −0.1 °C/sec in NEBuffer 2 (NEB) using a programmable thermocycler. The re-annealed PCR product was digested by 10 units of T7 endonuclease I (T7EI, NEB, Cat. No. M0302L) for 15 minutes at 37 °C. The reaction was stopped by the addition of 0.25 M EDTA solution, and the sample was placed on ice. The PCR products were analyzed on 2% agarose gel to quantify the intensity of the digested and undigested bands by ImageJ software or on High Sensitivity D1000 ScreenTape with TapeStation 2200 (Agilent Technologies). The percentage of nuclease-specific cleavage peak area in the summed band area (expressed as the fraction cleaved) was used to estimate the gene editing levels using the following equation^35^, % mutation = 100 × (1 − (1 − *f*)^1/2^), where *f* is the fraction cleaved.

### ssODN-mediated single nucleotide editing

One day prior to transfection, 0.3–0.5 × 10^6^ iPS cells were plated in recombinant laminin-511 E8 (2.4 μg/well, iMatrix-511, Nippi, Cat. No. 892012)-coated wells of 6-well plates. After collecting the cells, 0.5 × 10^6^ cells were transfected with 12 μg of ssODN (custom synthesized by FASMAC, 10 nmol scale, reverse-phase column purification grade, dissolved in MilliQ water with 12 μg/μL concentration) in 20 μL Primary P4 buffer using 4D-Nucleofector (Lonza) and the electroporation condition program CA-137. The ssODN sequences for transfection are shown in Supplementary Table 6. After electroporation, cells were transferred to 1.2 μg/well iMatrix-511-coated 12-well plates and grown in AK03N medium containing ROCK inhibitor Y-27632. To perform genome cleavage, 2 μM Dox and 1 μM Dex were added into the medium, and cells were cultured for 24–48 hours.

To check knock-in efficiency, genomic DNA was extracted from the transfected cells and analyzed by qPCR, RFLP and Sanger sequencing. qPCR was performed with specific primers according to the manufacturer’s instructions. The primer sequences for qPCR are shown in Supplementary Table 7.

The genomic regions around the sgRNA target site for the *DMD, ILF3*, and *HLA-A* genes were amplified by PCR. The sgRNA target sequences are shown in Supplementary Table 2. To check whether the designed restriction sites (AgeI, PstI or AvrII) were introduced via ssODN-mediated knock-in experiments, the PCR product was digested with the indicated restriction enzyme and analyzed by agarose gel electrophoresis or 2200 TapeStation (Agilent Technologies). The primer sequences for PCR are shown in Supplementary Table 5.

To determine the frequency of indel and HR, PCR products were cloned with TA-cloning. Picked colonies were boiled in water, used as templates for PCR amplification and enzymatically cleaned up by Exonuclease I (Cat. No. 2650 A, Takara) and Alkaline phosphatase (Shrimp) (Cat. No. 2660 A, Takara). The sequences of each clone were analyzed by using a BigDye Terminator v3.1 Cycle Sequencing Kit (Cat. No. 4337455, Thermo Fisher) and Applied Biosystems 3500xL Genetic Analyzers. Low quality sequence reads (i.e. no signal or mixed signals) were discarded, and the remaining multiple FASTA sequence files were combined by Unix “cat” command and aligned by “clustalo” algorithm using SeaView software. The logos of DNA and protein sequences were generated by WebLogo software (http://weblogo.threeplusone.com/create.cgi).

We isolated iPS cell subclones as previously described^36^. Briefly, ssODN-transfected iPS cells were treated with Y-27632 treatment for at least one hour and dissociated into single cells by TrypLE. Counted cells were plated at the density of 100–1,000 cells per one well of 6-well plate coated with iMatrix-511. Individual colonies were manually picked under EVOS FL microscope (Thermo Fisher) within a clean cabinet and genotyped by genomic PCR using the primers shown in Supplementary Table 5.

### Analysis of randomization patterns by Amplicon sequencing

The target region was PCR amplified with barcoding primers (DMD-MiSeq-Rd1-fwd1-9 and DMD-MiSeq-Rd2-rev1-9, shown in Supplementary Table 8) and then adapter primers (Multiplex P5 fwd and Multiplex P7 rev, shown in Supplementary Table 8) using a high-fidelity PCR enzyme. The resultant PCR products were gel-purified and quantified by the KAPA Library Quantification Kit for Illumina (Cat. no. KK4835, KAPA Biosystems). Each DNA sample was adjusted to 2 nM and denatured by freshly made 0.2 N NaOH solution for 5 min. The samples were further diluted to 12 pM, mixed with 4 pM of PhiX spike-in DNA (Cat. no. FC-110-3001, Illumina), and run on a single lane of HiSeq. 2500 (Illumina) by 1 × 100 bp sequencing reaction (>10 M reads). The generated FASTQ sequence files were filtered by FastQC program to remove low quality sequencing reads. The remaining sequencing reads were split based on the barcode indices by a custom shell script. Perfectly matched reads for the original sequence or ssODN template were separated, and the remaining reads, which include sequencing errors, were considered as indel sequences.

### Statistic analysis

To perform Student’s *t*-test, T.TEST function in Excel software was used with two tailed, non-paired, unequal variance analysis options. *p* < 0.05 was considered as statistically significant.

### Data availability

The datasets generated during the current study are available from the corresponding author on reasonable request.

### Ethical approval

No live vertebrates were used in this study. All the human cell lines used in this study are from publically available cell banks, and no isolation of *de novo* samples involved. Use of human iPS cells was approved by the Ethics Committee of the Department of Medicine at Kyoto University. All methods were performed in accordance with the approved guidelines.

## Electronic supplementary material

Supplementary information
