# Supplementary material for: Site-specific randomization of the endogenous genome by a regulatable CRISPR-Cas9 piggyBac system in human cells
Source: Sci Rep. 2018 Jan 10;8:310. doi: 10.1038/s41598-017-18568-4 (PMC5762678; doi:10.1038/s41598-017-18568-4)
Supplement: Supplementary file 1 — Supplementary information [file 41598_2017_18568_MOESM1_ESM.pdf]

Site-specific randomization of the endogenous genome by a regulatable CRISPR-Cas9 *piggyBac* system in human cells

Kentaro Ishida[1, 2], Huaigeng Xu[1], Noriko Sasakawa[1, 2], Mandy Siu Yu Lung[1], Julia Alexandra Kudryashev[3], Peter Gee[1], and Akitsu Hotta[1, 2, 4, *]

[1] Department of Life Science Frontiers, Center for iPS cell Research and Application (CiRA), Kyoto University, Kyoto, Japan.

[2] Core Center for iPS Cell Research, Research Center Network for Realization of Regenerative Medicine, Japan Agency for Medical Research and Development (AMED), Tokyo, Japan.

[3] Massachusetts Institute of Technology, Cambridge, Massachusetts, USA.

[4] Institute for integrated Cell-Material Sciences (iCeMS), Kyoto University, Kyoto, Japan.

* To whom correspondence should be addressed. Tel: +81-75-366-7056; Fax: +81-75-366-7074; Email: akitsu.hotta@cira.kyoto-u.ac.jp

Supplementary Methods

Supplementary Figures 1–11

Supplementary Tables 1–8

Supplementary References

Supplementary Methods

To construct the *piggyBac* vector backbone, 5' and 3' terminal repeats from *Trichoplusia ni* were synthesized as 3 gBlock fragments (IDT) (**Supplementary Table 2**) and inserted into the AatII-PvuII site of pUC19 to construct a pPV-synthesized plasmid. The PacI-HindIII fragment from PB-TetO2-RfA-IRESpuro-pA (PB-TAP, a kind gift from Dr. Knut Woltjen) was cloned into the SmaI-HindIII site of the PB-EF1α-EiP vector to construct the PB-TetO-GW-iP plasmid. Then, the NheI-PacI fragment was transferred to the NheI-PacI site of the pPV-synthesized vector to construct the pPV-TetO-GW-iP plasmid. Next, the rabbit hemoglobin poly A signal was amplified from the pCXLE-EGFP plasmid (a kind gift from Dr. Keisuke Okita) using primers pHL-PacI-rHBB-pA-IF-fw (5'-GTATACCTCGAGTTAAATTCACTCCTCAGGTGC-3') and pPV-PacI-rHBB-pA-IF-rev (5'-CGAGCTTGTTGGTTAATTAAGTCGAGGGATCTCCATAA-3') and inserted into the PacI site by In-Fusion cloning to construct the pPV-TetO-GW-iP-A plasmid. To construct the pPV-TetO-GW-iP-A-EF1α-rtTA-iH vector, we inserted the AsiSI-HincII fragment from PB-EF1α-rtTA-iH into the PacI-EcoRV site of the pPV-TetO-GW-iP-A vector. Simultaneously, the pPV-TetO-GW-iC-A vector was constructed by inserting a ApaI-PacI digested IRES-mCherry-pA fragment into the ApaI-PacI site of the pPV-TetO-GW-iP-A vector. Finally, the AsiSI-HincII fragment of the EF1α-rtTA-iH cassette was inserted into the PacI-EcoRV site of the pPV-TetO-GW-iC-A to construct the pPV-TetO-GW-iC-A-EF1α-rtTA-iH vector. Similarly, the AgeI-SphI fragment from pPV-EF1α-rtTA-iP-A was inserted into the AgeI-SphI site of pPV-TetO-GW-iC-A-EF1α-rtTA-iH to construct the pPV-TetO-GW-iC-A-EF1α-rtTA-iP vector.

Human codon optimized SpCas9 cDNA from pUC57-SphcCas9 was inserted into the SalI-XbaI site of the pENTR2B vector (Cat. No. A10463, Thermo Fisher) to construct the pENTR-SphcCas9 vector. Then, SpCas9 cDNA was inserted into the pPV-TetO-GW-iC-A-EF1α-rtTA-iP or pPV-TetO-GW-iC-A-EF1α-rtTA-iH vector by the Gateway LR Clonase II (Cat. No. 11791-020, Thermo Fisher Scientific). ERT2 cDNA was PCR amplified from pCR2.1-CreERT2 (kind gift from Dr. Kazutoshi Takahashi) by KOD-Plus-Neo (Cat. No. KOD-401, Toyobo) with the following primers; ER_pENTR_CAS9_Fwd: 5’-GGGCGGCGACTCCGGACTCGAGCCATCTGCTGGAGA-3’ and ER_pENTR_CAS9_Rev: 5’-TGGGGTCAGCTCCGGATCAAGCTGTGGCAGGGAAAC-3’. The ERT2 amplicon was inserted into the 3’ end of the pENTR-SphcCas9 at the BspEI restriction enzyme site by In-Fusion cloning to construct the pENTR-SphcCas9-ERT2. Then, SpCas9-ERT2 cDNA was inserted into the pPV-TetO-GW-iC-A-EF1α-rtTA-iP or pPV-TetO-GW-iC-A-EF1α-rtTA-iH vector by Gateway LR Clonase II. Human GR cDNA was PCR amplified from pPyCAG_cGR_IP with the following primers; Cas9-hGR-IF(BspEI)-fwd: 5'-GGGCGGCGACTCCGGAGCTGACGGAAGCGAAAATCCTGGTAACAAAACAATAG-3' and Cas9-hGR-IF(XbaI)-rev: 5'-GAAAGCTGGGTCTAGACTCACTTTTGATGAAACAGAAGT-3’. The hGR amplicon was inserted into the 3’ end of the pENTR-SphcCas9 at the BspEI-XbaI restriction enzyme site by In-Fusion cloning to construct the pENTR-SphcCas9-hGR. Then, SpCas9-GR cDNA was inserted into the pPV-TetO-GW-iC-A-EF1α-rtTA-iP or pPV-TetO-GW-iC-A-EF1α-rtTA-iH vector by the Gateway LR Clonase II reaction to construct the pPV-TetO-SphcCas9-GR-iC-A-EF1α-rtTA-iP (CRONUS-Puro, Addgene ID: 100596) or pPV-TetO-SphcCas9-GR-iC-A-EF1α-rtTA-iH (CRONUS-Hygro, Addgene ID: 100597) vector, respectively.

To construct a sgRNA-expressing pPV-H1-ccdB-EF1α-RiH *piggyBac* vector, the NheI-PacI fragment of the pHL-H1-ccdB-EF1α-RiH vector (Addgene ID: 60601) was inserted into the pPV-synthesized vector. Subsequently, each target sgRNA was inserted into the BamHI-EcoRI site of the pPV-H1-ccdB-EF1α-RiH vector (Addgene ID: 100598). The oligo DNA sequences for sgRNA cloning are shown in **Supplementary Table 3.** Human codon optimized *piggyBac* transposase cDNA (hcPBase) was custom synthesized by GenScript and cloned into pENTR2B vector. Then, hcPBase cDNA was inserted into the pHL-EF1α-GW-A vector by the LR Clonase II reaction to construct pHL-EF1α-hcPBase-A vector (Addgene ID: 100599).

Supplementary Figure Legend

Supplementary Figure 1: Effects of suppressing the NHEJ pathway on ssODN-mediated HR efficiency.

(**a**) Allele-specific primer design to distinguish original (WT) and genome-edited alleles for assessing knock-in efficiency by the ssODN donor template. The green primer sequence amplifies the original genomic allele only. The red primer sequence amplifies the genome-edited allele only. A common reverse primer was used for both. (**b**) Validation of the allele specific primers. WT and edited (KI) allele sequences were mixed at the indicated percentage and subjected by qPCR using the allele specific primer sets. Edited allele was readily detected quantitatively as low as 0.1% of the KI allele. (**c**) The indicated siRNA was transfected by RNAiMax lipofection reagents and on the next subsequent day, plasmid DNAs (expressing Cas9 or sgRNA) and the ssODN template were transfected by

lipofectamine 2000. Two days after transfection, the knock-down efficiency of each target gene was measured by qRT-PCR. White bar, siRNA control; black bar, cells treated with siRNA. (**d**) Effects of siRNAs against NHEJ factors on knock-in efficiency by ssODN were assessed by the allele-specific primers. We also tested a mixing of the 3 siRNAs (mix: KU70, KU80, and LIG4). Copy numbers of the edited alleles were normalized to that of *NANOG* allele. (**e**) At the time of transfection with Cas9, sgRNA and ssODN template, the indicated chemical compounds (L7: L755507, BA: Brefeldin A, YM: YM155) were added to the culture media. Two days after transfection, genomic DNA was isolated, and knock-in efficiency was evaluated by allele-specific qPCR.

Supplementary Figure 2: Characterization of the CRONUS iPS cells.
(**a**) Sanger sequencing of subclones confirmed successful knock-in in 10 clones out of 72 clones analyzed. Cas9 cleavage site is indicated by a blue triangle on top. (**b**) Parental 1383D2 iPS cells and CRONUS 1383D2 iPS cells were differentiated into embryoid bodies (EB) and the expression of the pluripotency marker NANOG gene was analyzed by qRT-PCR. The expression of NANOG relative to GAPDH was down-regulated after 10 days of EB formation, suggesting successful differentiation. (**c**) Experimental scheme for optimal duration of Dox and Dex treatment relative to the timing of ssODN donor electroporation. Green arrows indicate the culture duration with the presence of Dox and Dex. (**d**) Knock-in efficiency was measured by RFLP assay using AgeI restriction enzyme.

Supplementary Figure 3: Off-target analysis on *DMD* locus of the CRONUS iPS cells.
(**a**) Potential off-target sites in human genome (hg19) were predicted by web-based tool CRISPOR with CFD scores > 0.2. (**b**) CRONUS iPS cells (10 passages after establishment in the absent of Dox and Dex) were treated with Dox, Dex and ssODN for 24 hours, and each predicted site was examined by the T7EI assay. Note that on the OT1 site, we found that an AluYb9 retrotransposon was inserted in the iPS cells, and a poly A stretch of the AluYb9 element generated background cleaved bands by T7EI activity even without the induction of on-target cleavage (data not shown). No other sites showed detectable mutagenesis up to 24 hours of Dox and Dex treatment. Arrowheads indicate expected cleavage bands in the case of off-target mutagenesis.
(**c**) The same genomic DNA in Fig. S2b (10 passages without Dox/Dex treatment) was used to examine background genome cleavage at the *DMD* locus by Sanger sequencing. The PAM sequence is boxed, and the Cas9 cleavage site is indicated by the arrowhead.

Supplementary Figure 4: Off-target analysis on *ILF3* locus of the CRONUS iPS cells.
(**a**) Potential off-target sites in human genome (hg19) were predicted by by web-based tool CRISPOR with CFD scores > 0.2. (**b**) CRONUS iPS cells were treated with Dox, Dex and ssODN for 24 hours, and each predicted site was examined by the T7EI assay. Potential off-target sites showed detectable mutagenesis up to 24 hours of Dox and Dex treatment. Arrowheads indicate expected cleavage bands in the case of off-target mutagenesis.

Supplementary Figure 5: Off-target analysis on *HLA-A* locus of the CRONUS iPS cells.
(**a**) Potential off-target sites in human genome (hg19) were predicted by web-based tool CRISPOR with CFD scores > 0.2. (**b**) CRONUS iPS cells were treated with Dox, Dex and ssODN for 24 hours, and each predicted site was examined by the T7EI assay. Potential off-target sites showed detectable mutagenesis up to 24 hours of Dox and Dex treatment. Arrowheads indicate expected cleavage bands in the case of off-target mutagenesis. (**c**) The potential off-target sites in the non-targeting allele of *HLA-A* gene (A*32:01), and related *HLA-B* and *HLA-C* gene locus (on chr6) were examined by TA-cloning and Sanger sequencing. The numbers of sequenced *E.coli* clones are indicated to on the right.

Supplementary Figure 6: Breakdown of all the sequencing reads analyzed at the *DMD* gene locus in Fig. 5b.
Deleted nucleic acids are indicated by "-", and randomized dinucleic acids are indicated in bold and underlined. The gRNA-targeting sequence is indicated by the blue underline on the WT sequence clones, the PAM sequence is boxed, and the cleavage site is indicated by the arrowhead. The numbers of sequenced clones are indicated on to the right.

Supplementary Figure 7: Breakdown of all the sequence reads analyzed at the *HLA-A* gene locus in Fig. 6f.
Deleted nucleic acids are indicated by "-", and randomized dinucleic acids are indicated in bold and underlined. The gRNA- targeting sequence is indicated by the blue underline on the WT sequence clones, the PAM sequence is boxed, and the Cac9 cleavage site is indicated by the arrowhead. The numbers of sequenced clones are indicated to the on right hand.

Supplementary figures

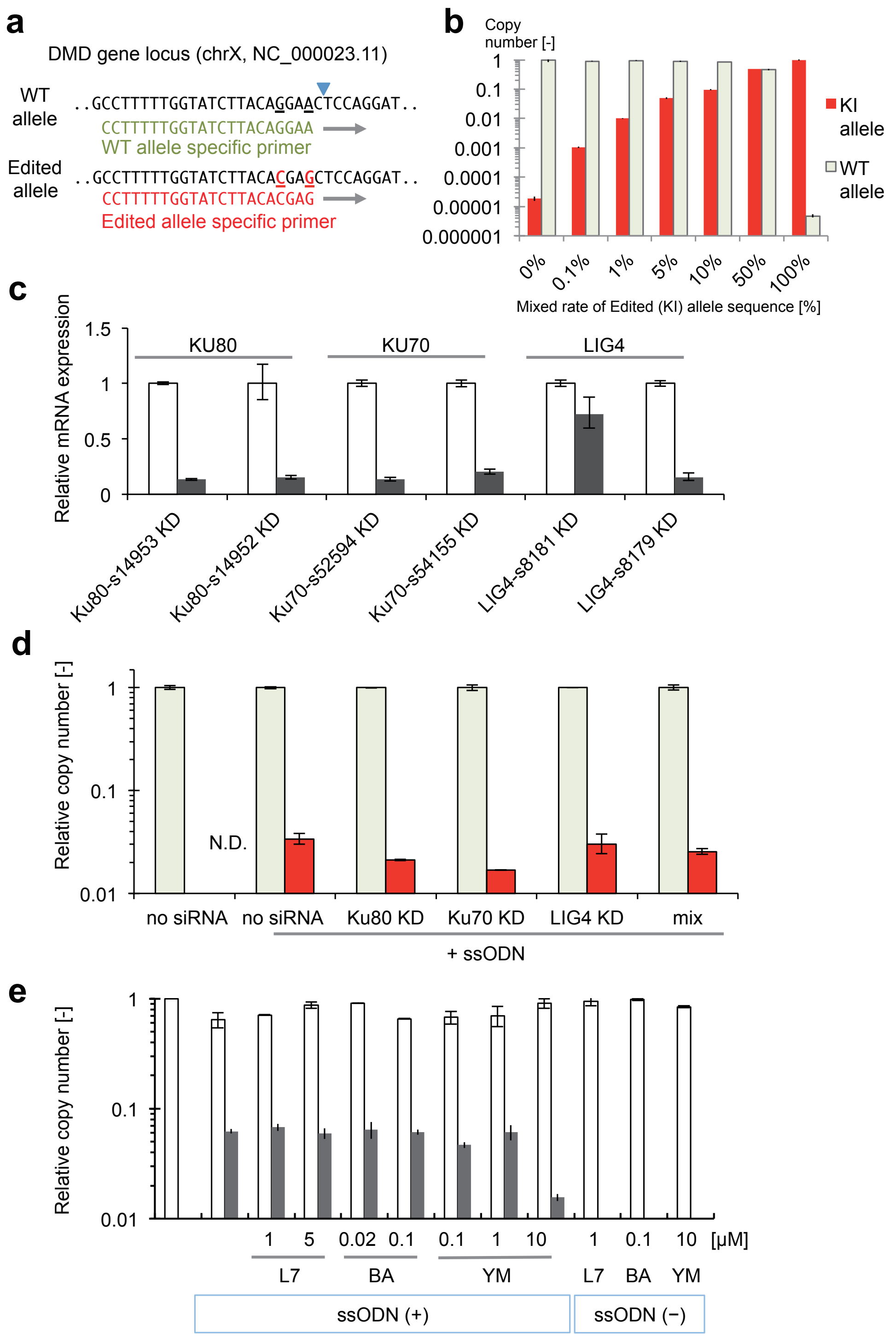

Supplementary Figure 1: Effects of suppressing the NHEJ pathway on ssODN-mediated HR efficiency.

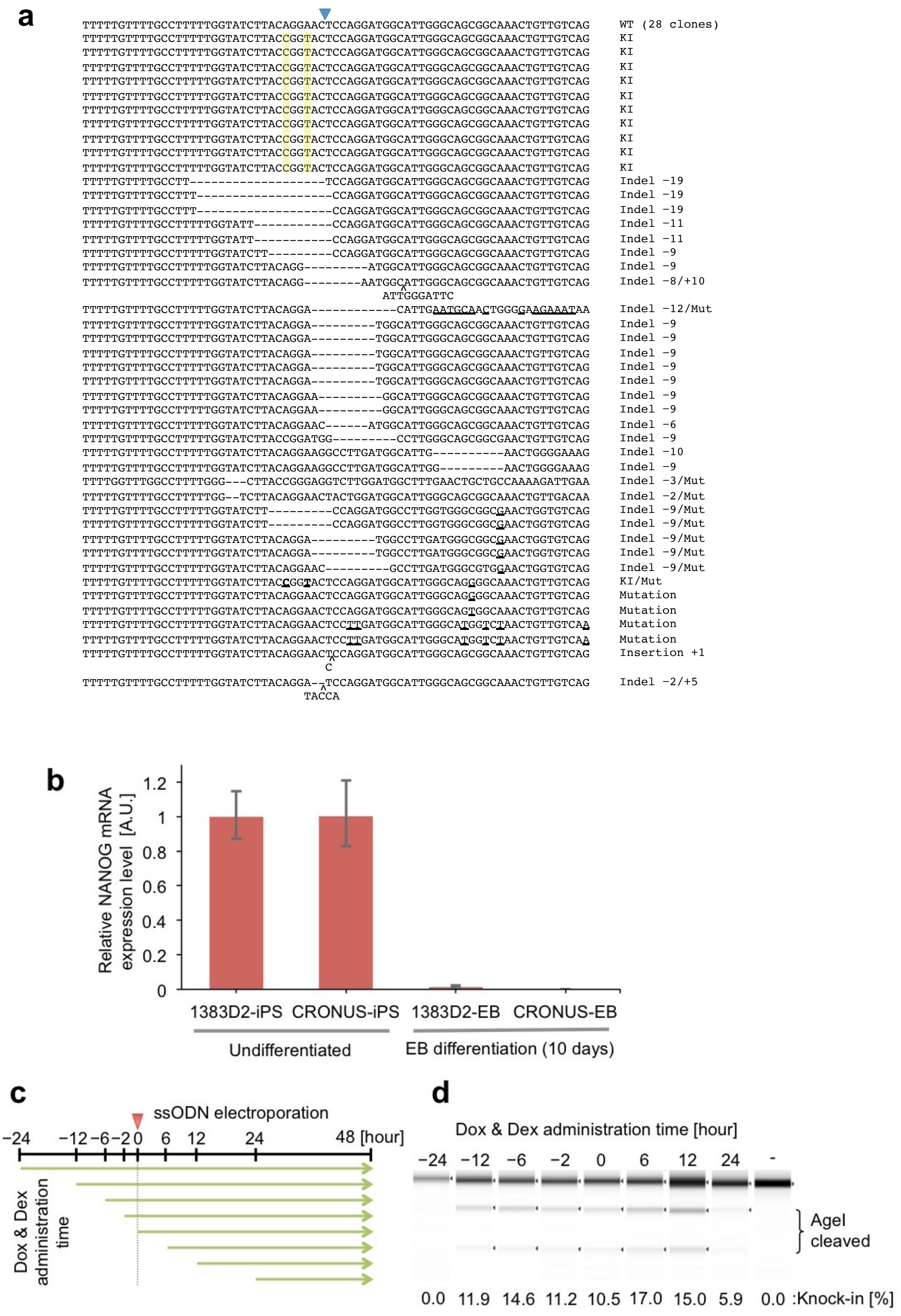

Supplementary Figure 2: Characterization of the CRONUS iPS cells.

a DMD potential off-target sites

		Position	Sequence	No. of mismatches	CFD off-target score
On-target	DMD	ChrX:31986622-31986644	TGGTATCTTACAGGAACTCCAGG	0	1.000
Potential off-target sites	POS#1	Chr14: 52643754-52643776	TGGTATCTTAaAGGaACTCCAGG	2	0.563
	POS#2	Chr2:73856067-73856089	TGGTgaCcTACAGGAACaCCAGG	4	0.416
	POS#3	Chr10:87439853-87439875	caGTATtTTgCAGGAACTCCAGG	4	0.327
	POS#4	Chr1:18055504-18055526	TGacATCTTACAGGgACTCCCGG	3	0.315
	POS#5	Chr2:141638398-141638420	atGTAaCTTACAGGAACTCaAGG	4	0.275
	POS#6	Chr3:133984249-133984271	TGGTgTCTTtCAGaAAtTCCAGG	4	0.222
	POS#7	ChrY:9969346-9969368	aGGTATgTTACAgGAACTCcTGG	4	0.217
	POS#8	Chr3:13117765-13117787	TGGgcaCTTACAGaAACTCCAGG	4	0.203
	POS#9	Chr11:93292265-93292287	gGGTAatTTACAGGAACTCtGGG	4	0.202
	POS#10	Chr12:104838770-104838792	aGGTAgCTTACAGgAAcTCCAGG	4	0.200

b

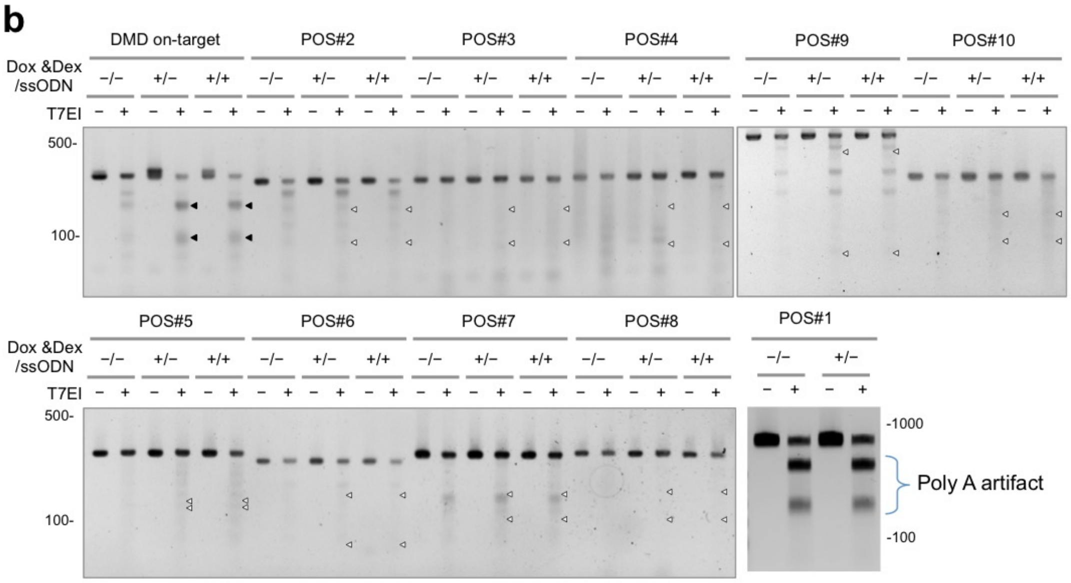

c

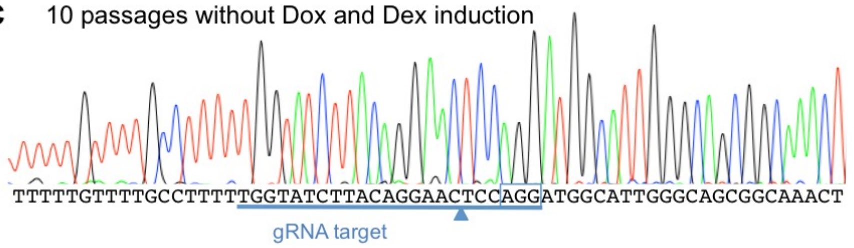

Supplementary Figure 3: Off-target analysis on *DMD* locus of the CRONUS iPS cells.

a

ILF3 potential off-target sites

		Position	Sequence	No. of mismatches	CFD off-target score
On-target	ILF3	Chr19:10798021-10798043	GGTCAGTTCTACAGCAACGGAGG	0	1.000
Potential off-target sites	POS#1	Chr4:134991647-134991669	tGTCAGTgCaACAGCAAtGGAGG	4	0.404
	POS#2	Chr3:38649263-38649285	GGaCAGaTCagCAGCAACGGAGG	4	0.348
	POS#3	Chr8:47955014-47955036	ccTCAGTcCTACAGCAAtGGAGG	4	0.317
	POS#4	Chr3:4839226-4839248	tGTCAGTTCTAaAaCAAaGGAGG	4	0.288
	POS#5	Chr9:90488795-90488817	GaTCAGcTCaACAGCAAaGGAGG	4	0.268
	POS#6	Chr5:61124989-61125011	GGTCAGTagTACAaCAACaGAGG	4	0.265
	POS#7	Chr17:7380141-7380163	GcTaAGTTCTgCAGCAACtGAGG	4	0.252
	POS#8	Chr2:113696236-113696258	GGcCAaaTCTACAGCAAtGGAGG	4	0.241
	POS#9	Chr11:1361284-1361306	GGTCAtTTCTAaAGCAACatAGG	4	0.238
	POS#10	Chr15:66415875-66415897	GGTtAcTTCTACgGCAACtGAGG	4	0.237
	POS#11	Chr2:140497471- 140497493	tGTCAtTTCcACAGCAACtGAGG	4	0.237
	POS#12	Chr2: 201730079-201730101	tGgCAGcTCTACAGCAACtGAGG	4	0.229

b

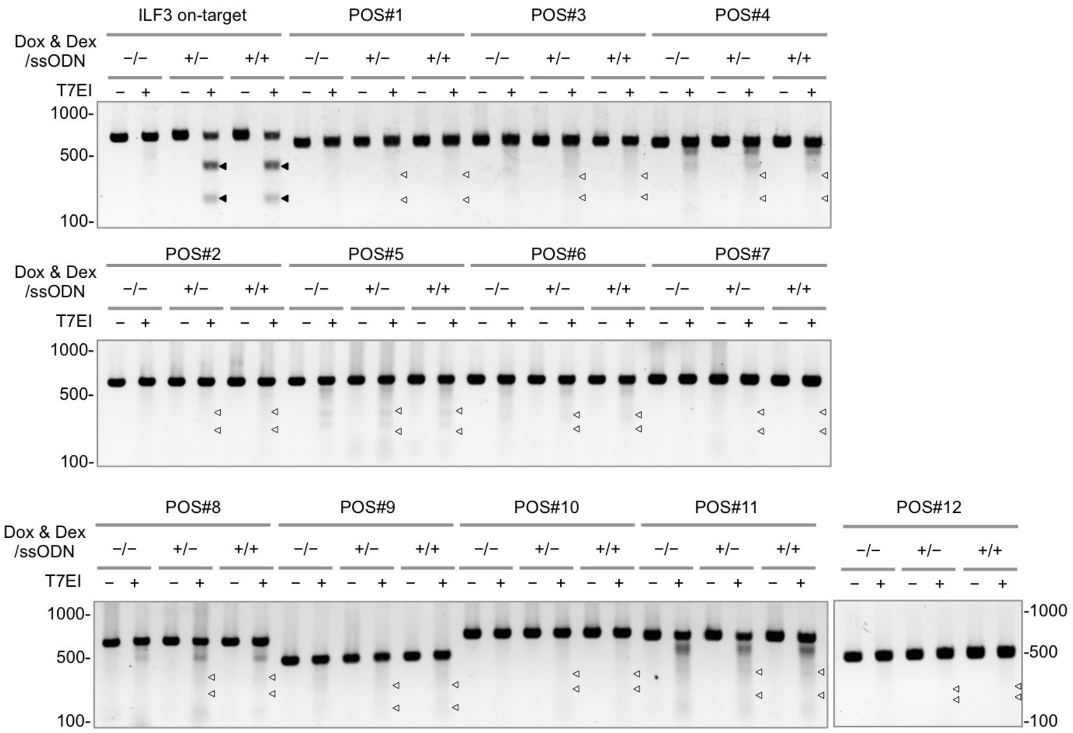

Supplementary Figure 4: Off-target analysis on *ILF3* locus of the CRONUS iPS cells.

a

HLA-A potential off-target sites

		Position	Sequence	No. of mismatches	CFD off-target score
On-target	HLA-A	Chr6:1292998-1293020	TACCACCAGTACGCCTACGACGG	0	1.000
Potential off-target sites	POS#1	Chr6:2836819-2836841	cAtaACCAGTACGCCTACGACGG	3	0.496
	POS#2	Chr3:10796161-10796183	TAaCACCAGaAaGCCTACaATGG	4	0.379
	POS#3	Chr6:2619570-2619592	TAtgACCAGTACGCCTACGACGG	2	0.344
	POS#4	Chr6:31324132-31324154	cAtgACCAGTACGCCTACGACGG	3	0.295
	POS#5	Chr7:57744158-57744180	TACCACCAcTACatCTACaAGGG	4	0.260
	POS#6	Chr9:101216417-101216439	TtCCACggGTACGCCTACGATGG	3	0.251
	POS#7	Chr6:1240444-1240466	TACCAgCAGgACGCCTACGACGG	2	0.250
	POS#8	Chr13:30045970-30045992	TACCcaCAGTAaGCCTACtAGGG	4	0.221

b

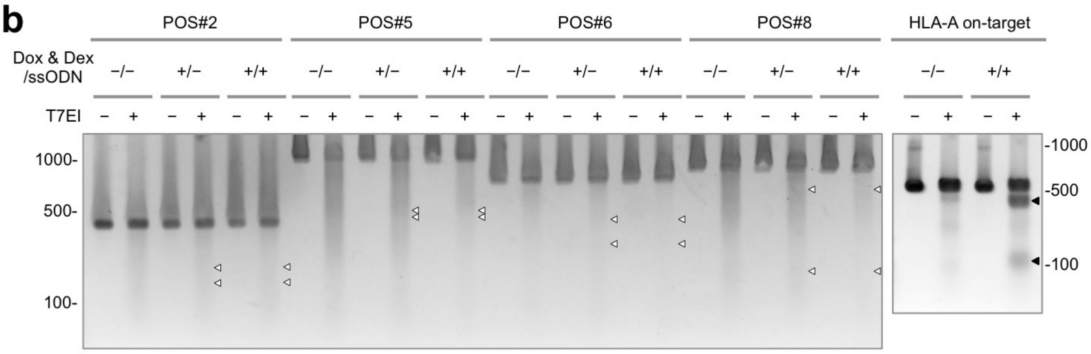

c

		Mutated / Analyzed
HLA-A*02:07 (On-target)	GTACCACCAGTACGCCTACGACGGCAA (gRNA target)	10 / 18
HLA-A*32:01	GTACCAgCAGgACGCCTACGACGGCAA	0 / 62
HLA-B*15:02	GTAtgACCAGTcCGCCTACGACGGCAA	0 / 39
HLA-B*51:01	GcAtaACCAGTACGCCTACGACGGCAA	0 / 14
HLA-C*08:01	GTAtaACCAGTtCGCCTACGACGGCAA	0 / 38
HLA-C*14:02	GTAtgACCAGTcCGCCTACGACGGCAA	0 / 67

Supplementary Figure 5: Off-target analysis on *HLA-A* locus of the CRONUS iPS cells.

	Sequence	No. of sequenced Clones
WT 76 clones	TTTTTGTTTTGCCTTTTTGGTATCTTACAGGAACTCCAGGATGGCATTGGGCAGCGGCAA	76
Indel or Mut 22 clones	CATTGGGCAGCGGCAAACTGTTGT------------CAGGATGGCATTGGGCAGCGGCAA	1
	TAA--------CCTTTTTGGTATCTTACAGGAAGTCCAGGATGGCATTGGGCAGCGGCAA	1
	TTTTTGTTTTGCCTTTTTGGTATCTT---------CCAGGATGGCATTGGGCAGCGGCAA	1
	TTTTTGTTTTGCCTTTTTGGTATCTT---------CCAGGATGGCATTGGGCAGCGGCAA	2
	TTTTTGTTTTGCCTTTTTGGTATCAT---------CCAGGATGGCATTGGGCAGCGGCAA	1
	TTTTTGTTTTGCCTTTTTGGTATCTTA---------CAGGATGGCATTGGGCAGCGGCAA	2
	TTTTTGTTTTGCCTTTTTGGTATCTTA---------CAGGAACGCATTGGGCAGCGGCAA	1
	TTTTTGTTTTGCCTTTTTGGTATCTTA---------CAGGATGGCATTGGGCAGCGGCAA	1
	TTTTTGTTTTGCCTTTTTGGTATCTTAC--------AGGAATGGCATTGGGCAGCGGCAA	1
	TTTTTGTTTTGCCTTTTTGGTATCTTACAGG---------ATGGCATTGGGCAGCGGCAA	1
	TTTTTGTTTTGCCTTTTTGGTATCTTACAGGAA---------GGCATTGGGCAGCGGCAA	1
	TTTTTGTTTTGCCTTTTTGGTATCTTACAGGAA---CAGGATGGCATTGGGCAGCGGCAA	1
	TTTTTGTTTTGCCTTTTTGGTATCTTACAGGAAC------ATGGCATTGGGCAGCGGCAA	2
	TTTTTGTTTTGCCTTTTTGGTATCTTACAGGAACTCCAGG--GG-ATTGGGCAGCGGCAA	1
	TTTTTGTTTTGCCTTTTTGGTATCTTCCAGGA---------TGGCATTGGGCAGCGGCAA	1
	TTTT-GTTTTGCCTTTTTGGTATCTTACAGGAACTCCAGGATGGCATTGGGCAGCGGCAA	1
	TTTGTTTTGCACCTTTTTGGTATCTTACAGGAACTCCAGGATGGCATTGGGCAGCGGCAA	1
	TTTTTGTTTTGCCTTTTTGGTATCTAACAGGAA**AC**CCAGGATGGCATTGGGCAGCGGCAA	1
	TTCAGGTATACCTGTAAGATATCTTACAGGTATCTCCAGGATGGCATTGGGCAGCGGCAA	1
Randomized 53 clones	TTTTTGTTTTGCCTTTTTGGTATCTTACAGGAA**AA**CCAGGATGGCATTGGGCAGCGGCAA	1
	TTTTTGTTTTGCCTTTTTGGTATCTTACAGGAA**AC**CCAGGATGGCATTGGGCAGCGGCAA	5
	TTTTTGTTTTGCCTTTTTGGTATCTTACAGGAA**AG**CCAGGATGGCATTGGGCAGCGGCAA	5
	TTTTTGTTTTGCCTTTTTGGTATCTTACAGGAA**AT**CCAGGATGGCATTGGGCAGCGGCAA	3
	TTTTTGTTTTGCCTTTTTGGTATCTTACAGGAA**CA**CCAGGATGGCATTGGGCAGCGGCAA	2
	TTTTTGTTTTGCCTTTTTGGTATCTTACAGGAA**CC**CCAGGATGGCATTGGGCAGCGGCAA	2
	TTTTTGTTTTGCCTTTTTGGTATCTTACAGGAA**CG**CCAGGATGGCATTGGGCAGCGGCAA	4
	TTTTTGTTTTGCCTTTTTGGTATCTTACAGGAA**GA**CCAGGATGGCATTGGGCAGCGGCAA	5
	TTTTTGTTTTGCCTTTTTGGTATCTTACAGGAA**GC**CCAGGATGGCATTGGGCAGCGGCAA	1
	TTTTTGTTTTGCCTTTTTGGTATCTTACAGGAA**GG**CCAGGATGGCATTGGGCAGCGGCAA	4
	TTTTTGTTTTGCCTTTTTGGTATCTTACAGGAA**GT**CCAGGATGGCATTGGGCAGCGGCAA	5
	TTTTTGTTTTGCCTTTTTGGTATCTTACAGGAA**TA**CCAGGATGGCATTGGGCAGCGGCAA	1
	TTTTTGTTTTGCCTTTTTGGTATCTTACAGGAA**TC**CCAGGATGGCATTGGGCAGCGGCAA	4
	TTTTTGTTTTGCCTTTTTGGTATCTTACAGGAA**TG**CCAGGATGGCATTGGGCAGCGGCAA	3
	TTTTTGTTTTGCCTTTTTGGTATCTTACAGGAA**TT**CCAGGATGGCATTGGGCAGCGGCAA	8

Supplementary Figure 6: Breakdown of all the sequencing reads analyzed at the *DMD* gene locus in Fig. 5b.

		No. of sequenced Clones
WT 58 clones	GCTTCCTCCGCGGGTACCACCAGTACGCCTACGACGGCAAGGATTACATCGCCCTGAAAG	58
Indel or Mut 27 clones	GCTTCCTCCGCGGGTA--AG	1
	GCTTCCTCCGCGGGTA---------------------------TTACATCGCCCTGAAAG	1
	GCTTCCTCCGCGGGTACCACC-----------GACGGCAAGGATTACATCGCCCTGAAAG	1
	GCTTCCTCCGCGGGTACCACCAG----------ACGGCAAGGATTACATCGCCCTGAAAG	1
	GCTTCCTCCGCGGGTACCACCAGT-----------GGCAAGGATTACATCGCCCTGAAAG	1
	GCTTCCTCCGCGGGTACCACCAGTAC--------------GGATTACATCGTCCTGAAAG	1
	GCTTCCTCCGCGGGTACCACCAGTAC----------GCAAGGATTACATCGCCCTGAAAG	1
	GCTTCCTCCGCGGGTACCACCAGTAC------GACGGCAAGGATTACATCGCCCTGAAAG	1
	GCTTCCTCCGCGGGTACCACCAGTAC------GACGGCAAGGATTACATCGCCCTGAACG	1
	GCTTCCTCCGCGGGTACCACCAGTAC------GACGGCGAGGATTACTTCGCCCTGAAAA	1
	GCTTCCTCCGCGGGTACCACCAGTACG------ACGGCAAGGATTACATCGCCCTGAAA-	1
	GCTTCCTCCGCGGGTACCACCAGTACGCCT-AGACGGCAAGGATTACATCGCCCTGAAAG	1
	GCTTCCTCCGCGGGTACCACCAGTACGCCT-CGACGGCAAGGATTACATCGCCCTGAAAG	1
	GCTTCCTCCGCGGGTACCACCAGTACGCCTA-------AAGGATTACATCGCCCTGAAAG	1
	GCTTCCTCC-CGGGTACCACCAGTACGCC**AG**CGACGGCAAGGATTACATCGCCCTGAAAG	1
	GCCTCCTCCGCGGGTACCAGCAGGACGCCTACGACGGCAAGGATTACATCGCCTTGAACG	6
	GCTTCCTCCACGGGTACCACCAGTAAGCC**GG**CGACGGCAAGGATTACATCGCCCTGAAAG	1
	GCTTCCTCCGCGAGTACCACCAGTACGCC**TC**CGACGGCAAGGATTACATCGCCCTGAAAG	1
	GCTTCCTCCGCGGGTACCACATCAGTACG**CC**AACGACGGCAAGGATTACTATCGCCCTGA	1
	GCTTCCTCCGCGGGTACCACCAGTACGCCTAGCGACGGCAAGGATTACATCGCCCTGAAA	1
	GCTTCCTCCGCGGGTACCACCAGTACTCC**TT**CTACGGCTGGGATTACATCGCCCTGAAAG	1
	GCTTCCTCCGCGGGTACCACCAGTACGCCTACGACGGCGAGGATTACATCGCCCTGAAAG	1
Randomized 67 clones	GCTTCCTCCGCGGGTACCACCAGTACGCC**AA**CGACGGCAAGGATTACATCGCCCTGAAAG	4
	GCTTCCTCCGCGGGTACCACCAGTACGCC**AG**CGACGGCAAGGATTACATCGCCCTGAAAG	3
	GCTTCCTCCGCGGGTACCACCAGTACGCC**AT**CGACGGCAAGGATTACATCGCCCTGAAAG	2
	GCTTCCTCCGCGGGTACCACCAGTACGCC**CA**CGACGGCAAGGATTACATCGCCCTGAAAG	3
	GCTTCCTCCGCGGGTACCACCAGTACGCC**CC**CGACGGCAAGGATTACATCGCCCTGAAAG	6
	GCTTCCTCCGCGGGTACCACCAGTACGCC**CG**CGACGGCAAGGATTACATCGCCCTGAAAG	4
	GCTTCCTCCGCGGGTACCACCAGTACGCC**CT**CGACGGCAAGGATTACATCGCCCTGAAAG	6
	GCTTCCTCCGCGGGTACCACCAGTACGCC**GA**CGACGGCAAGGATTACATCGCCCTGAAAG	4
	GCTTCCTCCGCGGGTACCACCAGTACGCC**GC**CGACGGCAAGGATTACATCGCCCTGAAAG	1
	GCTTCCTCCGCGGGTACCACCAGTACGCC**GG**CGACGGCAAGGATTACATCGCCCTGAAAG	3
	GCTTCCTCCGCGGGTACCACCAGTACGCC**GT**CGACGGCAAGGATTACATCGCCCTGAAAG	6
	GCTTCCTCCGCGGGTACCACCAGTACGCC**TC**CGACGGCAAGGATTACATCGCCCTGAAAG	9
	GCTTCCTCCGCGGGTACCACCAGTACGCC**TG**CGACGGCAAGGATTACATCGCCCTGAAAG	10
	GCTTCCTCCGCGGGTACCACCAGTACGCC**TT**CGACGGCAAGGATTACATCGCCCTGAAAG	6

Supplementary Figure 7: Breakdown of all the sequence reads analyzed at the *HLA-A* gene locus in Fig. 6f.

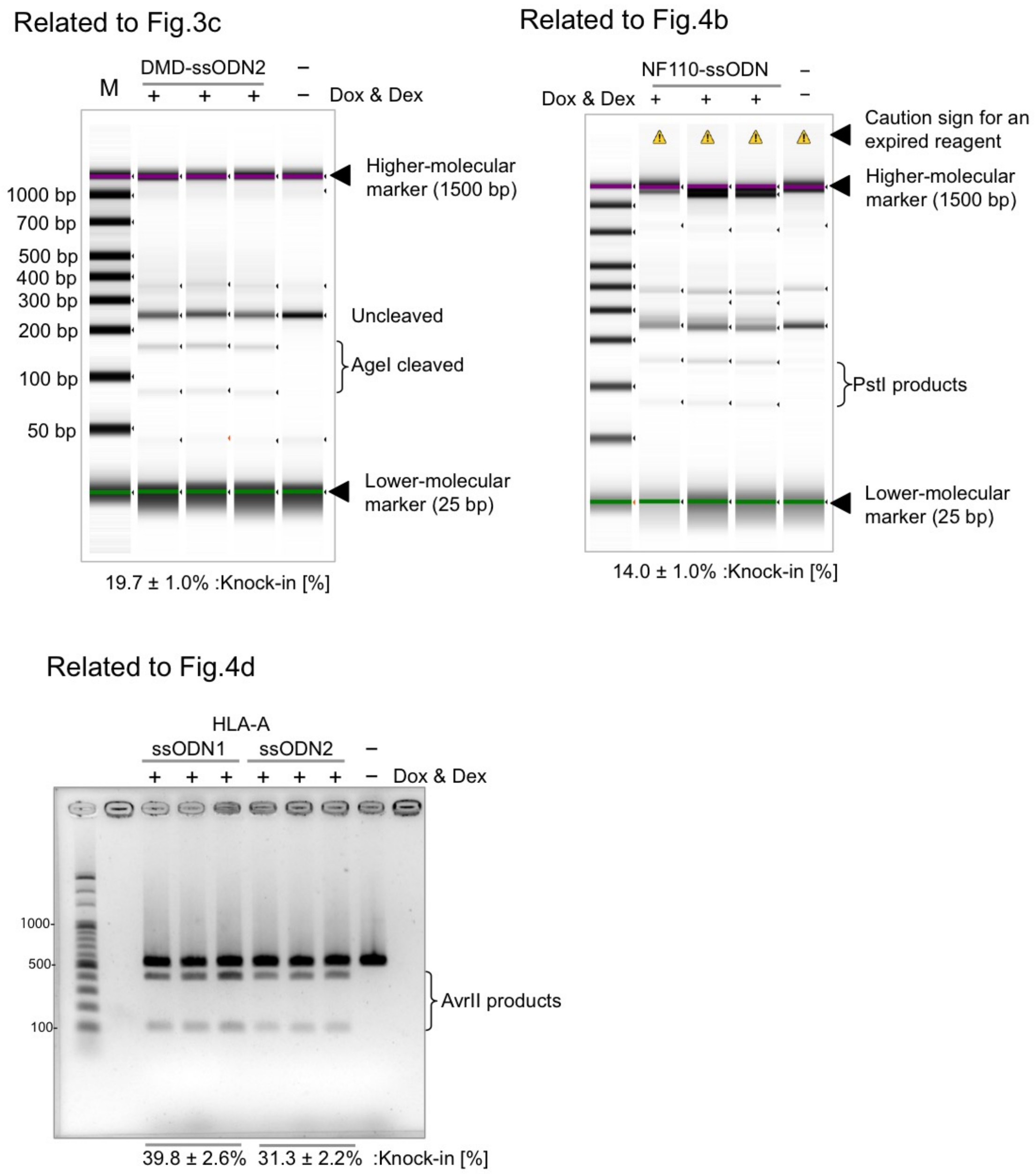

Supplementary Figure 8: Full-length gel images for Fig. 3c, 4b, and 4d.

Related to Fig.S3b

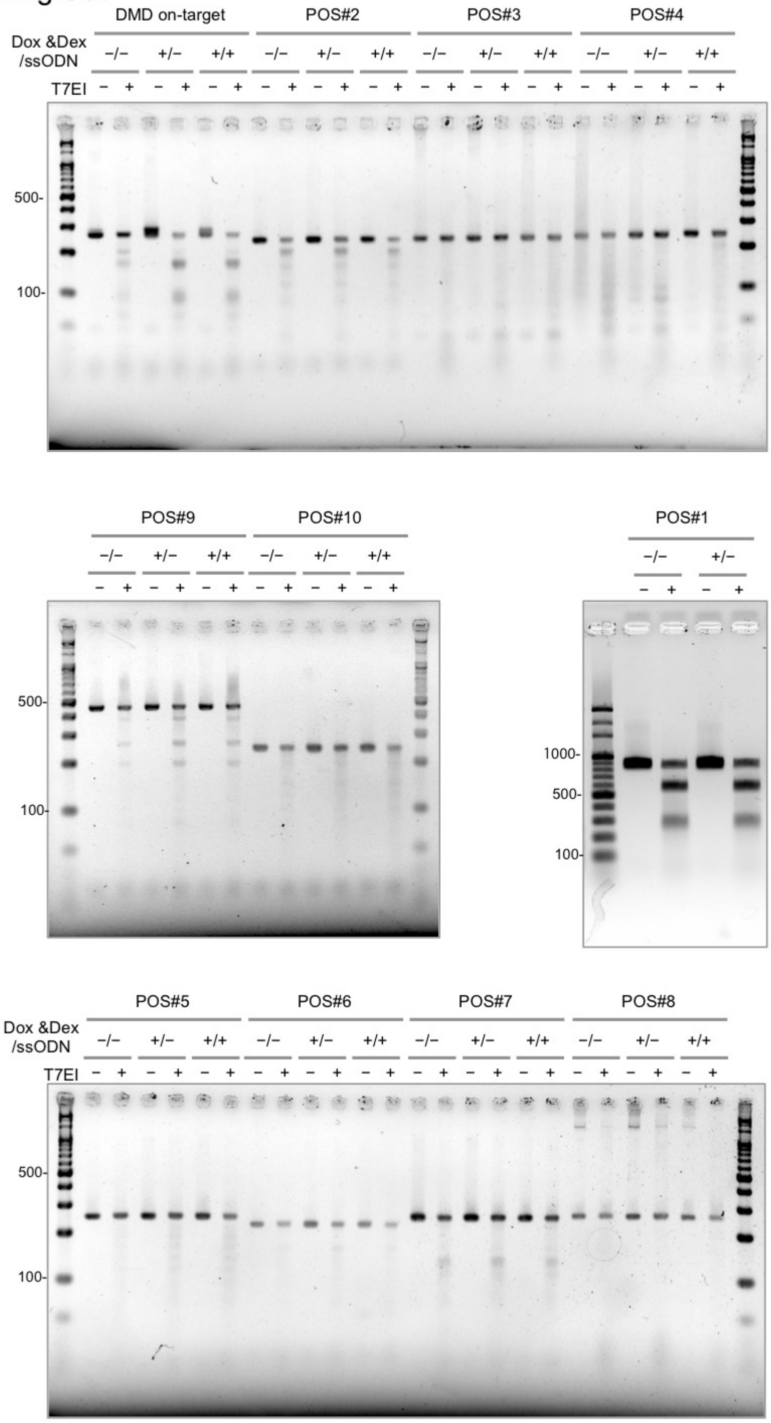

Supplementary Figure 9: Full-length gel images for Fig. S2b

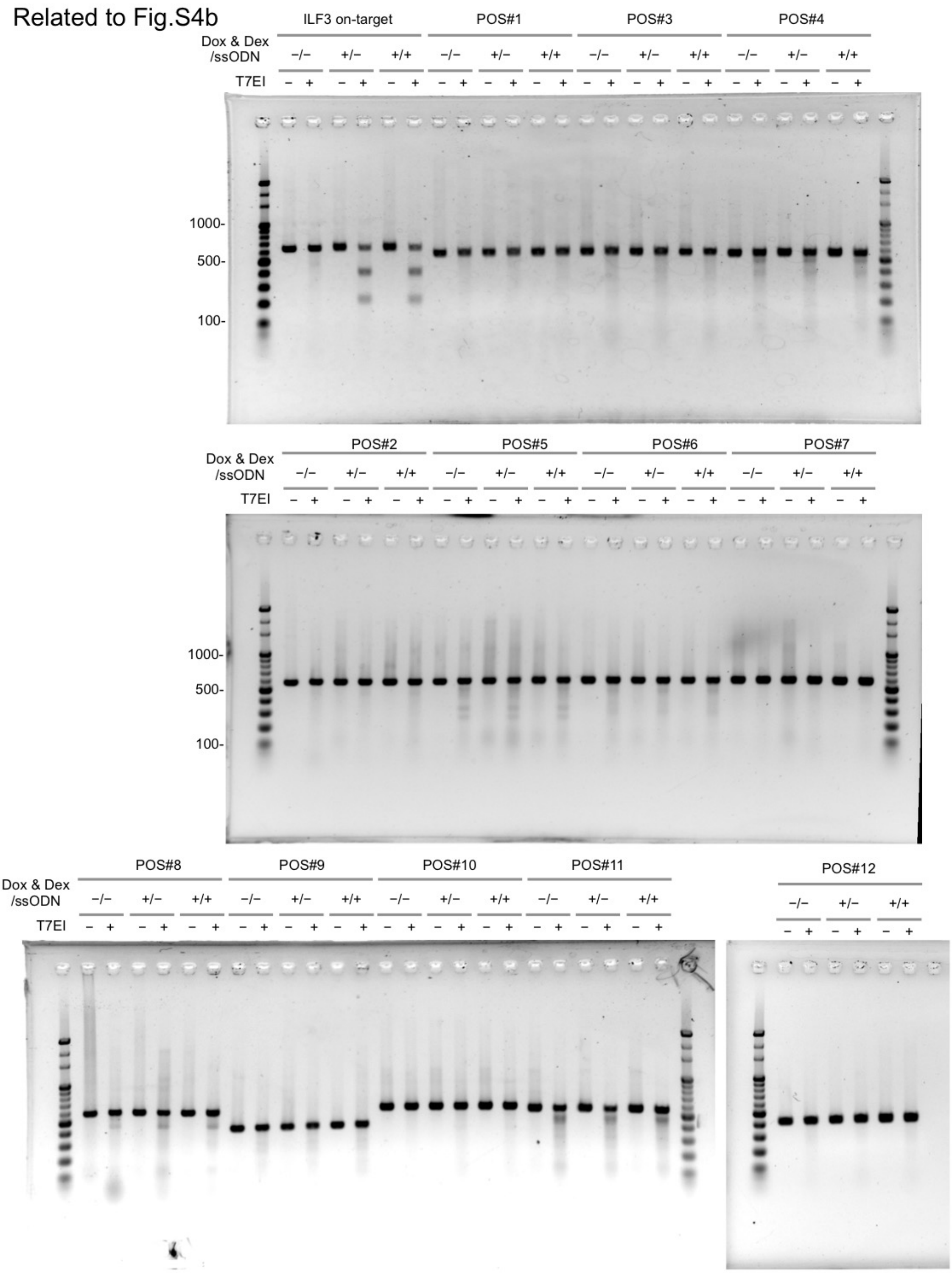

Supplementary Figure 10: Full-length gel images for Fig. S3b

Related to Fig. S5b

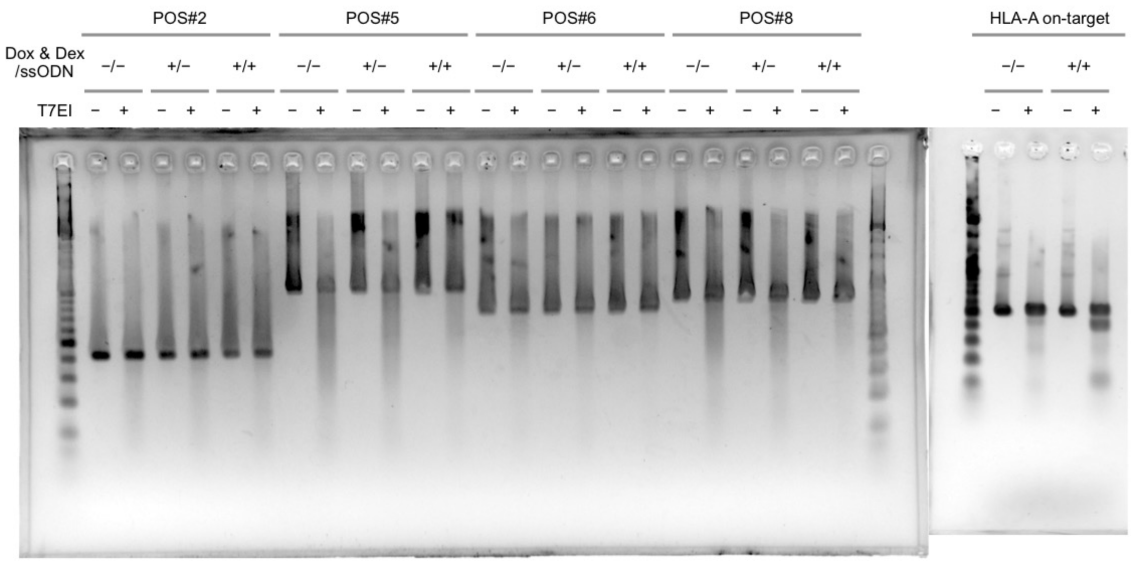

Supplementary Figure 11: Full-length gel images for Fig. S4b

Supplementary Tables

Supplementary Table 1: Various knock-in efficiencies on ssODN-mediated genome editing

Cell type	Gene	Transduction method of Nuclease	Knock-in [%]	Note	Ref.
K562	RSK2	ZFN electroporation as mRNA or DNA	22–32%		(1)
293FT	EMX1	SpCas9(D10A) lipofection as plasmid DNA	1.7–17%		(2)
293T	EMX1	SpCas9 electroporation as protein	22%		(3)
			31%	G2/M arrest with Nocodazole	
293T	CXCR4	SpCas9 electroporation as protein	6%		(3)
			38%	G2/M arrest with Nocodazole	
293T	DYRK1	SpCas9 electroporation as protein	4%		(3)
			25%	G2/M arrest with Nocodazole	
Neonatal fibroblasts	EMX1	SpCas9 electroporation as protein	N.D.		(3)
			1.3%	G2/M arrest with Nocodazole	
293T	RBM20	SpCas9 lipofection as plasmid DNA	~1%		(4)
HCT116	Mutant EGFP	SpCas9 electroporation as plasmid DNA	~3%		(5)
293T	RBM20	SpCas9 lipofection as plasmid DNA	25%		(6)
HEK293	EXM1	SpCas9 electroporation as protein	28.5%		(7)
HEK293	CXCR4	SpCas9 electroporation as protein	55%		(7)
K562	CXCR4	SpCas9 electroporation as protein	39.5%		(7)
U2OS	SUFU	TALEN electroporation as plasmid DNA	20–50%	Chemically modified ssODN	(8)
hESC	EMX1	SpCas9(D10A) electroporation as plasmid DNA	~0.4%		(2)
hESC	GATA6	Dox-inducible SpCas9 knock-in into AAVS1	15%	iCRISPR method	(9)
hESC	APOE	Dox-inducible SpCas9 knock-in into AAVS1	3%	iCRISPR method	(9)
hESC	EMX1	SpCas9 electroporation as protein	N.D.		(3)
			1.6%	G2/M arrest with Nocodazole	
hESC	SNCA	ZFN electroporation as plasmid DNA	0.83%		(10)
hESC	SNCA	ZFN electroporation as plasmid DNA	0.41%		(10)
iPSC	GATA6	Dox-inducible SpCas9 knock-in into AAVS1	8%	iCRISPR method	(9)
iPSC	APOE	Dox-inducible SpCas9 knock-in into AAVS1	2%		(9)
iPSC	SOD1	SpCas9 electroporation as plasmid DNA	0.35%		(11)
			3.13%	NHEJ inhibitor L77705	
iPSC	Lentiviral BFP	SpCas9 electroporation as plasmid DNA	1.2%	Conversion to eGFP	(12)
iPSC	APP	SpCas9 electroporation as plasmid DNA	0.3–6.7%	"CORRECT" method	(13)
iPSC	PSEN	SpCas9 electroporation as plasmid DNA	1.2–3.2%	"CORRECT" method	(13)
iPSC	HBB	SpCas9 nucleofection as plasmid DNA	0.1–0.3%		(14)
			0.07–5.2%	Sib-selection	
iPSC	HBB	SpCas9 by HDAd/EBV vector	24–67.9%	Sib-selection	(14)
iPCS	TAZ	Dox-inducible SpCas9 by *piggyBac*	38%		(15)
iPCS	DMD	Dox-inducible SpCas9-ER by *piggyBac*	19.7%	"CRONUS" method	This study
iPCS	ILF3	Dox-inducible SpCas9-ER by *piggyBac*	14%	"CRONUS" method	This study
iPCS	HLA-A	Dox-inducible SpCas9-ER by *piggyBac*	39.8%	"CRONUS" method	This study

Supplementary Table 2: List of gBlock sequences used for construction of the pPV *piggyBac* vector

Oligo ID	gBlock sequence
gBlock11-PV-3'TR-5'TR	GAAAAGTGCCACCTGACGTCATCTGTTAACATTATACGCGTTTAACCCTAGAAAGATAATCATATTGTGACGTACGTTAAAGATAATCATGCGTAAAATTGACGCATGTGTTTTATCGGTCTGTATATCGAGGTTTATTTATTAATTTGAATAGATATTAAGTTTTATTATATTTACACTTACATACTAATAATAAATTCAACAAACAATTTATTTATGTTTATTTATTTATTAAAAAAAAACAAAAACTCAAAATTTCTTCTATAAAGTAACAAAACTTTTAAACATTCTCTCTTTTACAAAAATAAACTTATTTTGTACTTTAAAAACAGTCATGTTGTATTATAAAATAAGTAATTAGCTTAACCTATACATAATAGAAACAAATTATACTTA
gBlock12-PV-3'TR-5'TR	CCTATACATAATAGAAACAAATTATACTTATTAGTCAGTCAGAAACAACTTTGGCACATATCAATATTATGCTCTGCTAGCGATATCTGTAAAACGACGGCCAGTTCTAGACTTAAGCTTCATGGTCATAGCTGTTTCCTGCTCGAGTTAATTAACCAACAAGCTCGTCATCGCTTTGCAGAAGAGCAGAGAGGATATGCTCATCGTCTAAAGAACTACCCATTTTATTATATATTAGTCACGATATCTATAACAAGAAAATATATATATAATAAGTTATCACGTAAGTAGAACATGAAATAACAATA
gBlock13-PV-3'TR-5'TR	ATCACGTAAGTAGAACATGAAATAACAATATAATTATCGTATGAGTTAAATCTTAAAAGTCACGTAAAAGATAATCATGCGTCATTTTGACTCACGCGGTCGTTATAGTTCAAAATCAGTGACACTTACCGCATTGACAAGCACGCCTCACGGGAGCTCCAAGCGGCGACTGAGATGTCCTAAATGCACAGCGACGGATTCGCGCTATTTAGAAAGAGAGAGCAATATTTCAAGAATGCATGCGTCAATTTTACGCAGACTATCTTTCTAGGGTTAATACGTATAATACATATGATTCAGCTGCATTAATGAATC

Supplementary Table 3: List of oligo DNAs for sgRNA cloning

Oligo ID	gRNA target sequence (under-line indicates PAM sequence)	Sequence (under-line indicates gRNA spacer)
DMD-sgRNA1-fwd	TGGTATCTTACAGGAACTCC AGG	GAGACCACTTGGATCCGGGTATCTTACAGGAACTCCGTTTTAGAGCTAGAAATAGCA
gRNA-NF110-Ex17-SA-2-fwd	GGTCAGTTCTACAGCAACGG AGG	GAGACCACTTGGATCCGGTCAGTTCTACAGCAACGGGTTTTAGAGCTAGAAATAGCA
HLA-A-02:07-Exon3-Sp-gRNA-1-fwd	TACCACCAGTACGCCTACGA CGG	GAGACCACTTGGATCCGACCACCAGTACGCCTACGAGTTTTAGAGCTAGAAATAGCA
sgRNA-Universal-rev		GCCCGGGTTTGAATTCAAAAAAAGCACCGACTCGGTGCCACTTTTTCAAGTTGATAACGGACTAGCCTTATTTTAACTTGCTATTTCTAGCTCTAA

Supplementary Table 4: List of siRNA sequences

Target gene	Cat. No.	siRNA sequence
LIG4	s8181	CAUUGAACCUGGUAAUUCUTT
LIG4	s8179	CCGUGAAUAUGAUUGCUAUTT
XRCC5 (KU80)	s14953	CCGCUGAGGUGACAACAAATT
XRCC5 (KU80)	s14952	CUCCAUUCCUGGUAUAGAATT

XRCC6 (KU70)	s5455	GAGUGAAGAUGAGUUGACATT
XRCC6 (KU70)	s52594	GAUCCAGGUUUGAUGCUCATT

Supplementary Table 5: List of primers for PCR experiments (T7EI, RFLP, Sanger)

Primer name	Sequence	Note
T7E1-DMD-F	TACAACTGCATGTGGTAGCACACTG	DMD-gRNA target
T7E1-DMD-R	TTCTGTCTGACAGCTGTTTGCAGAC	DMD-gRNA target
T7E1-NF110-Ex17-Fw	GCCCCATTGTTGTCATCAGC	ILF3-gRNA target
T7E1-NF110-Ex17-Rv	AGCTTCTGCCACTCCGTG	ILF3-gRNA target
NF110-ssODN-Fw	TCAGGACCCATGGCTGCCG	ILF3-gRNA target
NF110-ssODN-Rv	GGGACTGGTAGCCCGAGC	ILF3-gRNA target
HLA-A-be-ex1-fwd	TTGGGGATTCCCCAACTCC	1st nested primer
HLA-A-in7-rev	GTCCACTGTTCCGCCCAA	1st nested primer
HLA-A0207-ex2-fwd	GGTCCGGAGTATTGGGACGG	2nd nested primer
HLA-A0207-ex3-rev	TCTCAACTGCTCCGCCACAT	2nd nested primer
DMD_POS#1-Fwd	TATATGGAAGCGTGGTCTGTGATAATT	DMD-gRNA off target
DMD_POS#1-Rev	TCCCTTTTATCAGCTAGGGCTCTTA	DMD-gRNA off target
DMD_POS#2-fwd	TGCTGTGATTCTAGGGACTGC	DMD-gRNA off target
DMD_POS#2--rev	ACTCTGAATCCAAGAGAAAACCCA	DMD-gRNA off target
DMD_POS#3-fwd	TCCCACGTCAGGCCTATACT	DMD-gRNA off target
DMD_POS#3-rev	GAGTTGGACACTGGTGGGAG	DMD-gRNA off target
DMD_POS#4-fwd	GAAGGTCCTCATGGCCCATC	DMD-gRNA off target
DMD_POS#4-rev	GGTATGGACCAGACTAGGGGT	DMD-gRNA off target
DMD_POS#5-fwd	GGGAGAAAAGTGGCTAGTTGC	DMD-gRNA off target
DMD_POS#5-rev	TGGTGGCAAGGGAAGTTGTT	DMD-gRNA off target
DMD_POS#6-fwd	AGTTTCTCTGGCATGTATGAAAGTG	DMD-gRNA off target
DMD_POS#6-rev	TCCCTTTTATCAGCTAGGGCTCTTA	DMD-gRNA off target
DMD_POS#7-fwd	TCTCAGTTTCTCTCTGCCATTGT	DMD-gRNA off target
DMD_POS#7-rev	GTTGGGAGTTCCATGCCCTT	DMD-gRNA off target
DMD_POS#8-fwd	GATGTCTGGAGTGGGAAGCA	DMD-gRNA off target
DMD_POS#8-rev	CTGGACTTACCCCTTGGCAG	DMD-gRNA off target
DMD_POS#9-fwd	TCAATTATACCTCAACAAAGCTGAAAA	DMD-gRNA off target
DMD_POS#9-rev	CGAAAAACTTTACTGGCAATTGTAGT	DMD-gRNA off target
DMD_POS#10-fwd	AGGAAGGGATGTTAAAAGTTACTTGG	DMD-gRNA off target

DMD_POS#10-rev	GACAGTGATAGTGATATATTTACTGGG	DMD-gRNA off target
ILF3_POS#1-fwd	TGGACCCTTGTGAGCTTAGG	ILF3-gRNA off target
ILF3_POS#1-rev	TGGTAGCTCTTTTGCCAGCA	ILF3-gRNA off target
ILF3_POS#2-fwd	TGCTGGTCTGGAGTCATGTG	ILF3-gRNA off target
ILF3_POS#2-rev	CTTACCTGCCAGGACCCTTC	ILF3-gRNA off target
ILF3_POS#3-fwd	AGCAGGTCAGACATTAAACAGGT	ILF3-gRNA off target
ILF3_POS#3-rev	ATGCCCCTGGGTGATTGTG	ILF3-gRNA off target
ILF3_POS#4-fwd	TGCCTTAAGTGCATCAGTTCAC	ILF3-gRNA off target
ILF3_POS#4-rev	GGGGATAGATGAAACAAGATAGGCA	ILF3-gRNA off target
ILF3_POS#5-fwd	AAGATCTGTTGGTCATCAATGAATGA	ILF3-gRNA off target
ILF3_POS#5-rev	TCAAAACTTTGCCTGTTATTGCCT	ILF3-gRNA off target
ILF3_POS#6-fwd	TTCAGATTTGCCTAGGATTGAGGA	ILF3-gRNA off target
ILF3_POS#6-rev	TGTGACCCAGGCATTACTAGC	ILF3-gRNA off target
ILF3_POS#7-fwd	AACAAAACCCCAGACACCCA	ILF3-gRNA off target
ILF3_POS#7-rev	TGTGGCCACTCTGTCCTCA	ILF3-gRNA off target
ILF3_POS#8-fwd	GTACACCCTGTAAGCTCAGCA	ILF3-gRNA off target
ILF3_POS#8-rev	ACGTATAAGTTGCGACAGCTCT	ILF3-gRNA off target
ILF3_POS#9-fwd	TGACACCATTGTGCTCCAGC	ILF3-gRNA off target
ILF3_POS#9-rev	CCTGGTCATGGCTCATGGTT	ILF3-gRNA off target
ILF3_POS#10-fwd	ACATATAATCTGCAGGAGGGCTG	ILF3-gRNA off target
ILF3_POS#10-rev	ATGTTGTCACGCGCCTCC	ILF3-gRNA off target
ILF3_POS#11-fwd	GGTGACAAAGAAAGAGATGTTAGTAGC	ILF3-gRNA off target
ILF3_POS#11-rev	TTCTCCTTTTGCCTGTCATCA	ILF3-gRNA off target
ILF3_POS#12-fwd	CCACTAGCGTCAGATGGCTT	ILF3-gRNA off target
ILF3_POS#12-rev	ACTACCACCACATTCTGCGG	ILF3-gRNA off target
HLA-A_POS#1-fwd	CAGGATACTCGTGACGCGT	HLA-A-gRNA off target
HLA-A_POS#1-rev	ACACACGCGAAACATCCCAA	HLA-A-gRNA off target
HLA-A_POS#2-fwd	ATTTATGAGACATCCGCCTCCC	HLA-A-gRNA off target
HLA-A_POS#2-rev	GTGTTATCCAAGGACGCAGAGG	HLA-A-gRNA off target
HLA-A_POS#3-fwd	CAATCAGCGTCTCCGCAGT	HLA-A-gRNA off target
HLA-A_POS#3-rev	ATGCTAACAGGAACGCAGACA	HLA-A-gRNA off target
HLA-A_POS#3-fwd-2	CCGGAGAGAGCCCCAGTC	HLA-A-gRNA off target
HLA-A_POS#3-rev-2	TCCATTCAAGGGAGGGCGATA	HLA-A-gRNA off target
HLA-A_POS#4-fwd	CCAGTTCGTGAGGTTCGACA	HLA-A-gRNA off target

HLA-A_POS#4-rev	GACATTCTAGCGCTGATCCCAT	HLA-A-gRNA off target
HLA-A_POS#5-fwd	AAAGATTAGAACGTCTGCAATGCC	HLA-A-gRNA off target
HLA-A_POS#5-rev	GCCCAGCTCGATAAATCCTTTC	HLA-A-gRNA off target
HLA-A_POS#6-fwd	TTTAGACATACGGGATATGGGCC	HLA-A-gRNA off target
HLA-A_POS#6-rev	TGCAATCCTAGGGCCTGC	HLA-A-gRNA off target
HLA-A_POS#7-fwd	GGTCCGGAGTATTGGGACGG	HLA-A-gRNA off target
HLA-A_POS#7-rev	TCTCAACTGCTCCGCCACAT	HLA-A-gRNA off target
HLA-A_POS#8-fwd	TATTACCACACGGGTGCATCATGG	HLA-A-gRNA off target
HLA-A_POS#8-rev	TTAGGTCACAGGAACATCCGC	HLA-A-gRNA off target
hNANOG-Fw	TGAACCTCAGCTACAAACAG	NANOG qRT-PCR
hNANOG-Rv	TGGTGGTAGGAAGAGTAAAG	NANOG qRT-PCR

Supplementary Table 6: List of ssODNs for knock-in experiments

ID of ssODN	Sequence	Note
DMD-ssOligo1-ES<br>(DMD-ssODN1)	GACATGGGGCTTCATTTTTGTTTTGCCTTTTTGGTATCTTAC<br>ACGAGCTCCAGGATGGCATTGGGCAGCGGGCAGCGGCAG<br>CGGCAAACTGTTGTCAGAA	
DMD-ssOligo1-ES-Ag<br>eI (DMD-ssODN2 or<br>ssODN-AgeI)	AAAAAGACATGGGGCTTCATTTTTGTTTTGCCTTTTTGGTA<br>TCTTACCGGTACTCCAGGATGGCATTGGGCAGCGGCAAAC<br>TGTTGTCAGAACATTGAAT	AgeI site
NF110-PstI-ssODN<br>(NF110-ssODN)	GCCTGACCCACTGCCTCCCTGTTTAGGTCAGTTCTACTGCA<br>GCGGAGGGCATTCTGGGAATGCCAGTGGCGGTGGCGGCG<br>GGGGCGGTGGTGGCTCCTCC	PstI site
ssODN-HLA-A0207-e<br>x3-1bp<br>(HLA-ssODN1)	CTGCGACGTGGGGTCGGACTGGCGCTTCCTCCGCGGGTAC<br>CACCAGTACGCCTAGGACGGCAAGGATTACATCGCCCTGA<br>AAGAGGACCTGCGCTCTTGG	AvrII site
ssODN-HLA-A0207-e<br>x3-2bp<br>(HLA-ssODN2)	CTGCGACGTGGGGTCGGACTGGCGCTTCCTCCGCGGGTAC<br>CACCAGTACGCCTAGGACTGCAAGGATTACATCGCCCTGA<br>AAGAGGACCTGCGCTCTTGG	AvrII site
DMD-ssODN-ex45-N<br>N-170125<br>(DMD-ssODN-NN)	AAAAAGACATGGGGCTTCATTTTTGTTTTGCCTTTTTGGTA<br>TCTTACAGGAA**NN**CCAGGATGGCATTGGGCAGCGGCAAA<br>CTGTTGTCAGAACATTGAAT	Randomized
DMD-ssODN-ex45-N<br>N-170303-2<br>(DMD-ssODN-NN2)	AAAAAGACATGGGGCTTCATTTTTGTTTTGCCTTTTTGGTA<br>TCTTACAG**NN**A**NN**CCAGGATGGCATTGGGCAGCGGCAAA<br>CTGTTGTCAGAACATTGAAT	Randomized
DMD-ssODN-ex45-N<br>N-170303-3<br>(DMD-ssODN-NN3)	AAAAAGACATGGGGCTTCATTTTTGTTTTGCCTTTTTGGTA<br>TCTTACAGGAA**NNCNN**GGATGGCATTGGGCAGCGGCAAA<br>CTGTTGTCAGAACATTGAAT	Randomized
DMD-ssODN-ex45-N<br>N-170303-4	AAAAAGACATGGGGCTTCATTTTTGTTTTGCCTTTTTGGTA<br>TCTTACAG**NN**A**NN**C**NN**GGATGGCATTGGGCAGCGGCAAA	Randomized

(DMD-ssODN-NN4)	CTGTTGTCAGAACATTGAAT	
DMD-ssODN-ex45-NN-170303-5 (DMD-ssODN-NN5)	AAAAAGACATGGGGCTTCATTTTTGTTTTGCCTTTTTGGTA TCTTACAG**NN**A**NN**C**NN**G**NN**TGGCATTGGGCAGCGGCAAA CTGTTGTCAGAACATTGAAT	Randomized
ssODN-HLA-A0207-ex3-NN-170125 (HLA-SSODN-NN)	CTGCGACGTGGGGTCGGACTGGCGCTTCCTCCGCGGGTAC CACCAGTACGCC**NN**CGACGGCAAGGATTACATCGCCCTGA AAGAGGACCTGCGCTCTTGG	Randomized

Supplementary Table 7: List of primers for genomic copy number qPCR experiments

Primer name	Sequence	Note
DMD normal qPCR Fw	CCTTTTTGGTATCTTACAGGAA	Normal allele specific
DMD Exon skip qPCR Fw	CCTTTTTGGTATCTTACACGAG	Edited allele specific
DMD normal qPCR Rv	TTTCTTCCCCAGTTGCATTC	Common reverse
hNANOG-Fwd	CTTCACTTCCCAGGTGCAA	Endogenous control
hNANOG-Rev	AGGCTAGCCAACATGAGGAA	Endogenous control
hACTB-Fwd	CCAACCGCGAGAAGATGA	Endogenous control
hACTB-Rev	CCAGAGGCGTACAGGGATAG	Endogenous control

Supplementary Table 8: List of primers for Hiseq experiments

Primer name	Sequence	Note
DMD-MiSeq-Rd1-fwd1-AGTC	CTCTTTCCCTACACGACGCTCTTCCGATCTagtcAATAAAAAGACATGGGGCTTCA	1st PCR
DMD-MiSeq-Rd2-rev1-AGTC	CTGGAGTTCAGACGTGTGCTCTTCCGATCTagtcCTGGCATCTGTTTTTGAGGA	1st PCR
DMD-MiSeq-Rd1-fwd2-GTCA	CTCTTTCCCTACACGACGCTCTTCCGATCTgtcaAATAAAAAGACATGGGGCTTCA	1st PCR
DMD-MiSeq-Rd2-rev2-GTCA	CTGGAGTTCAGACGTGTGCTCTTCCGATCTgtcaCTGGCATCTGTTTTTGAGGA	1st PCR
DMD-MiSeq-Rd1-fwd3-TCAG	CTCTTTCCCTACACGACGCTCTTCCGATCTtcagAATAAAAAGACATGGGGCTTCA	1st PCR
DMD-MiSeq-Rd2-rev3-TCAG	CTGGAGTTCAGACGTGTGCTCTTCCGATCTtcagCTGGCATCTGTTTTTGAGGA	1st PCR
DMD-MiSeq-Rd1-fwd4-CAGT	CTCTTTCCCTACACGACGCTCTTCCGATCTcagtAATAAAAAGACATGGGGCTTCA	1st PCR
DMD-MiSeq-Rd2-rev4-CAGT	CTGGAGTTCAGACGTGTGCTCTTCCGATCTcagtCTGGCATCTGTTTTTGAGGA	1st PCR
DMD-MiSeq-Rd1-fwd5-ATGC	CTCTTTCCCTACACGACGCTCTTCCGATCTatgcAATAAAAAGACATGGGGCTTCA	1st PCR
DMD-MiSeq-Rd2-rev5-ATGC	CTGGAGTTCAGACGTGTGCTCTTCCGATCTatgcCTGGCATCTGTTTTTGAGGA	1st PCR
DMD-MiSeq-Rd1-fwd6-TGCA	CTCTTTCCCTACACGACGCTCTTCCGATCTtgcaAATAAAAAGACATGGGGCTTCA	1st PCR

DMD-MiSeq-Rd2-rev6-TGCA	CTGGAGTTCAGACGTGTGCTCTTCCGATCTtgcaCTGGCATCTGTTTTTGAGGA	1st PCR
DMD-MiSeq-Rd1-fwd7-GCAT	CTCTTTCCCTACACGACGCTCTTCCGATCTgcatAATAAAAAGACATGGGGCTTCA	1st PCR
DMD-MiSeq-Rd2-rev7-GCAT	CTGGAGTTCAGACGTGTGCTCTTCCGATCTgcatCTGGCATCTGTTTTTGAGGA	1st PCR
DMD-MiSeq-Rd1-fwd8-CATG	CTCTTTCCCTACACGACGCTCTTCCGATCTcatgAATAAAAAGACATGGGGCTTCA	1st PCR
DMD-MiSeq-Rd2-rev8-CATG	CTGGAGTTCAGACGTGTGCTCTTCCGATCTcatgCTGGCATCTGTTTTTGAGGA	1st PCR
DMD-MiSeq-Rd1-fwd9-AACG	CTCTTTCCCTACACGACGCTCTTCCGATCTaacgAATAAAAAGACATGGGGCTTCA	1st PCR
DMD-MiSeq-Rd2-rev9-AACG	CTGGAGTTCAGACGTGTGCTCTTCCGATCTaacgCTGGCATCTGTTTTTGAGGA	1st PCR
Multiplex P5 fwd	AATGATACGGCGACCACCGAGATCTACACTCTTTCCCTACACGACGCTC	2nd PCR
Multiplex P7 rev	CAAGCAGAAGACGGCATACGAGATGTGACTGGAGTTCAGACGTGTGCTC	2nd PCR

Supplementary References

1. Chen,F., Pruett-Miller,S.M., Huang,Y., Gjoka,M., Duda,K., Taunton,J., Collingwood,T.N., Frodin,M. and Davis,G.D. (2011) High-frequency genome editing using ssDNA oligonucleotides with zinc-finger nucleases. *Nat Methods*, **8**, 753–755.
2. Ran,F.A., Hsu,P.D., Lin,C.-Y., Gootenberg,J.S., Konermann,S., Trevino,A.E., Scott,D.A., Inoue,A., Matoba,S., Zhang,Y., *et al.* (2013) Double nicking by RNA-guided CRISPR Cas9 for enhanced genome editing specificity. *Cell*, **154**, 1380–1389.
3. Lin,S., Staahl,B.T., Alla,R.K. and Doudna,J.A. (2014) Enhanced homology-directed human genome engineering by controlled timing of CRISPR/Cas9 delivery. *Elife*, **4**.
4. Miyaoka,Y., Chan,A.H., Judge,L.M., Yoo,J., Huang,M., Nguyen,T.D., Lizarraga,P.P., So,P.-L. and Conklin,B.R. (2014) Isolation of single-base genome-edited human iPS cells without antibiotic selection. *Nat. Methods*, **11**, 291–3.
5. Bialk,P., Rivera-Torres,N., Strouse,B. and Kmiec,E.B. (2015) Regulation of Gene Editing Activity Directed by Single-Stranded Oligonucleotides and CRISPR/Cas9 Systems. *PLoS One*, **10**, e0129308.
6. Nguyen,D.P., Miyaoka,Y., Gilbert,L.A., Mayerl,S.J., Lee,B.H., Weissman,J.S., Conklin,B.R. and Wells,J.A. (2016) Ligand-binding domains of nuclear receptors facilitate tight control of split CRISPR activity. *Nat. Commun.*, **7**, 12009.
7. Richardson,C.D., Ray,G.J., DeWitt,M.A., Curie,G.L. and Corn,J.E. (2016) Enhancing homology-directed genome editing by catalytically active and inactive CRISPR-Cas9 using asymmetric donor DNA. *Nat. Biotechnol.*, **34**, 339–344.
8. Renaud,J.-B., Boix,C., Charpentier,M., De Cian,A., Cochennec,J., Duvernois-Berthet,E., Perrouault,L., Tesson,L., Edouard,J., Thinard,R., *et al.* (2016) Improved Genome Editing Efficiency and Flexibility Using Modified Oligonucleotides with TALEN and CRISPR-Cas9 Nucleases. *Cell Rep.*, **14**, 2263–2272.
9. González,F., Zhu,Z., Shi,Z.D., Lelli,K., Verma,N., Li,Q. V. and Huangfu,D. (2014) An iCRISPR platform for rapid, multiplexable, and inducible genome editing in human pluripotent stem cells. *Cell Stem Cell*, **15**, 215–226.
10. Soldner,F., Laganière,J., Cheng,A.W., Hockemeyer,D., Gao,Q., Alagappan,R., Khurana,V., Golbe,L.I., Myers,R.H., Lindquist,S., *et al.* (2011) Generation of isogenic pluripotent stem cells differing exclusively at two early onset Parkinson point mutations. *Cell*, **146**, 318–331.
11. Yu,C., Liu,Y., Ma,T., Liu,K., Xu,S., Zhang,Y., Liu,H., La Russa,M., Xie,M., Ding,S., *et al.* (2015) Small molecules enhance crispr genome editing in pluripotent stem cells. *Cell Stem Cell*, **16**, 142–147.
12. Flynn,R., Grundmann,A., Renz,P., Haenseler,W., James,W.S., Cowley,S.A. and Moore,M.D. (2015) CRISPR-mediated genotypic and phenotypic correction of a chronic granulomatous disease mutation in human

iPS cells. *Exp. Hematol.*, 10.1016/j.exphem.2015.06.002.
13. Paquet,D., Kwart,D., Chen,A., Sproul,A., Jacob,S., Teo,S., Olsen,K.M., Gregg,A., Noggle,S. and Tessier-Lavigne,M. (2016) Efficient introduction of specific homozygous and heterozygous mutations using CRISPR/Cas9. *Nature*, **533**, 1–18.
14. Li,C., Ding,L., Sun,C.-W., Wu,L.-C., Zhou,D., Pawlik,K.M., Khodadadi-Jamayran,A., Westin,E., Goldman,F.D. and Townes,T.M. (2016) Novel HDAd/EBV Reprogramming Vector and Highly Efficient Ad/CRISPR-Cas Sickle Cell Disease Gene Correction. *Sci. Rep.*, **6**, 30422.
15. Wang,G., Yang,L., Grishin,D., Rios,X., Ye,L.Y., Hu,Y., Li,K., Zhang,D., Church,G.M. and Pu,W.T. (2017) Efficient, footprint-free human iPSC genome editing by consolidation of Cas9/CRISPR and piggyBac technologies. *Nat Protoc.*, **12**, 88–103.
